# Effects of multiple cell regulators on curli gene expression in *Escherichia coli*

**DOI:** 10.1128/jb.00281-25

**Published:** 2025-11-12

**Authors:** Maryia Ratnikava, Olga Lamprecht, Victor Sourjik

**Affiliations:** 1 Max Planck Institute for Terrestrial Microbiology and Center for Synthetic Microbiology (SYNMIKRO) 28310 , Marburg, Germany; Geisel School of Medicine at Dartmouth, Hanover, New Hampshire, USA

**Keywords:** curli, transcriptional control, c-di-GMP, motility, RpoS, bimodality

## Abstract

Curli amyloid fibers are the key protenacious component of the extracellular matrix in *Escherichia coli*. The regulation of curli expression is highly complex and depends on multiple environmental responses. While many genetic determinants of curli production are known, previous studies have been conducted under various conditions and using different strains of *E. coli* or *Salmonella*. Furthermore, while the expression of curli genes in an *E. coli* population is known to be bimodal, the origins of this bimodality are only partially understood. Here, we systematically investigated the role of various cellular factors in the expression of the curli structural genes *csgBA* at the single-cell level in planktonic *E. coli* culture. We observed that multiple factors involved in the regulation of stress response, cell motility, cell physiology and metabolism, maintenance of DNA architecture, and second messenger signaling either promote or repress the expression of *csgBA* genes. We further elucidated which regulators act upstream of the master transcription factor CsgD and which are crucial for the bimodality of curli gene expression. Overall, this study provides an overview of the regulation of curli gene expression in planktonic *E. coli* culture in the absence of any microenvironmental gradients.

**IMPORTANCE:**

The transition from a solitary planktonic lifestyle to a multicellular biofilm is a complex developmental process involving multiple changes in bacterial cell physiology. For *Enterobacteriaceae*, a critical step in this process is the production of curli amyloid fibers, the main component of their extracellular matrix. A commitment to express curli genes already occurs in a subpopulation of planktonically growing *Escherichia coli* cells. Here, we investigated how this activation depends on multiple stress response factors, global regulators of gene expression, and the second messenger cyclic-di-GMP. We demonstrated that bimodal expression of curli structural genes in planktonic cultures requires an interplay between several transcription factors and chromosome-organizing proteins but not second messenger signaling.

## INTRODUCTION

Most bacteria are able to produce extracellular matrix that promotes formation of multicellular structures, giving protection against adverse environmental factors (1–3). Curli are the major proteinaceous component of the extracellular matrix produced by *Escherichia coli* (4–8). They are highly stable to degradation, thus supporting environmental cell persistence and cell stress survival (5, 6, 9). The formation of curli is known to be triggered by a combination of suboptimal growth conditions and environmental stresses, such as slow growth, nutrient starvation, low temperatures, low osmolarity, etc. (2, 10–15).

In *E. coli*, genes required for curli formation are organized in two oppositely transcribed operons, *csgBAC* and *csgDEFG* (2, 12, 15). They are separated by a 754 bp long intergenic non-coding region, which is one of the largest and most heavily regulated in *E. coli* (5, 16), enabling responses to multiple environmental conditions (8, 11, 13, 15, 17). The *csgBAC* operon mainly controls production of curli fibers, while the *csgDEFG* operon encodes a master curli regulator CsgD and accessory proteins required for assembly and secretion of the curli structural subunits CsgB and CsgA (5, 9, 18, 19). CsgD activates expression of curli structural genes as well as its own expression by binding to multiple sites within the intergenic *csg* region (9, 20). The expression of curli genes depends on the general stress response regulator RpoS, and it further depends on multiple transcriptional factors, small RNAs (sRNAs), and second messenger bis-(3′–5′)-cyclic di-GMP (c-di-GMP) (2, 5, 14, 15, 17, 21–31). A number of transcription factors have been reported to bind within the *csg* intergenic region and collaboratively modulate expression of curli genes from σ^70^- and σ^S^-dependent promoters (16, 23, 27, 32, 33). These include regulators of metabolic transitions, two-component signaling, flagellar gene expression, and maintenance of DNA architecture (IHF and H-NS) (2, 8, 16, 33–37).

Intracellular c-di-GMP concentration, another key regulator of curli gene expression, is itself under complex control by a set of diguanylate cyclases (DGCs) and phosphodiesterases (PDEs) (29, 38, 39). In total, 14 DGCs have been identified in non-pathogenic *E. coli* strains, including two degenerate proteins that lack the catalytic function (39, 40). Curli gene expression is primarily modulated by DgcE and DgcM (28, 29, 31, 41, 42). Elevated c-di-GMP levels promote expression of curli by derepressing MlrA, the transcriptional activator of *csgD* (28, 31). Other active DGCs, including DgcO and DgcP, as well as the degenerate DGC CsrD, have been shown to regulate CsgD activity in *E. coli* (39, 43).

Expression of curli within a population is restricted to a group of cells, resulting in formation of curli-producing and non-producing cells (44–48). Heterogeneous production of curli in macrocolony and submerged biofilms could be driven by the presence of microenvironmental and metabolic gradients (44, 49–51). However, bimodal expression of both *csgD* (44) and *csgB* (47) promoters was also observed in an isogenic well-stirred planktonic culture of *E. coli*. Despite the absence of pronounced cell clumping in cultures grown with vigorous shaking, both the fraction of *csgBA*-expressing *E. coli* cells and their expression levels at high aeration were similar to those observed in submerged or macrocolony biofilms (47). This suggested that the emergence of bimodality is an inherent property of curli gene activation and not the consequence of spatial biofilm structure, and that curli gene expression in *E. coli* is not repressed by high aeration.

Here, we systematically assessed how multiple cellular regulators and DGCs contribute to the expression of *csgBA* genes in such unstructured planktonic *E. coli* culture. We confirmed involvement of some but not the other putative curli regulators under our growth conditions and demonstrated impact of several regulators that were not previously shown to control curli gene expression. Furthermore, we classified which regulators act upstream of *csgD* and uncovered a set of regulators crucial for bimodality of expression of curli structural genes in the absence of microenvironmental gradients. Our study provides a systematic overview of curli regulation in planktonic *E. coli* culture.

## MATERIALS AND METHODS

### Bacterial strains and plasmids

All strains and plasmids used in this study are listed in Table S1. An RpoS^+^ derivative of *E. coli* W3110 (52) that was engineered to encode a chromosomal transcriptional superfolder green fluorescent protein (sfGFP) reporter downstream of the *csgA* gene (VS1146) (45) was used as the wild-type (WT) strain.

Majority of gene deletions were obtained with the help of P1 phage transduction as described previously (53), with some modifications. Strains of the Keio collection (54) were used as donors for the transfer of the kanamycin resistance cassette, which disrupted the coding sequence leaving intact only start codon and last 21 nucleotides (except for *dgcO* deletion, where first 342 and last 21 nucleotides were retained). Deletion of *dgcN* led to the in-frame addition of the 240 nucleotides encoding for a part of Tn5 transposase to the upstream-located *yfiR* gene. In the case of *csgD*, the deletion strain was constructed using the lambda red recombination method, with the first 200 nucleotides and last 30 nucleotides being kept to avoid possible disruption of the common curli gene regulatory region. In the strains that showed differences in curli expression from WT (except for Δ*ihfA*), the kanamycin resistance cassette was eliminated using flippase (Flp) recombinase as described previously (55) to confirm absence of polar effects of the cassette (56). Obtained gene deletions were confirmed by PCR with primers flanking the corresponding genes (Table S2) and in selected cases additionally by Sanger sequencing.

For complementation analysis of strains deficient in curli expression, *csgD* gene was cloned into the pTrc99A vector by restriction and ligation (57) and transformed in *E. coli* cells using the transformation and storage solution (TSS) method as described previously (58), with modifications.

### Growth conditions

To analyze levels of *csgBA* expression and fraction of curli-positive cells by flow cytometry, *E. coli* cultures were inoculated from lysogeny broth (LB) agar plates (10 g tryptone, 5 g yeast extract, 5 g NaCl, 15 g Bacto Agar per liter; pH 7.0) supplemented with 50 µg/mL kanamycin where necessary and grown in 5 mL of LB medium at 37°C and 200 rpm in 100 mL flasks in a rotary shaker overnight (14–16 h). Obtained cultures were diluted 1:100 in 5 mL of tryptone broth (TB) medium (10 g tryptone, 5 g NaCl per liter; pH 7.0) and grown at 30°C in a rotary shaker at 200 rpm in 100 mL flasks for a time required to detect maximum *csgBA* expression levels (22–25 h for WT, 12–17 h for Δ*dgcI*, and 17–30 h for other strains). For complementation analysis, cultures were supplemented with 100 µg/mL ampicillin and indicated levels of inducer isopropyl β-d-1-thiogalactopyranoside (IPTG).

To analyze reporter expression and growth curves using plate reader, *E. coli* overnight cultures grown as above were diluted 1:100 in TB medium and incubated at 30°C in INFINITE 200 PRO plate reader (Tecan Group Ltd., Switzerland) for 24–25 h.

### Flow cytometry

Aliquots of 40–70 µL of bacterial cultures were mixed with 2 mL of phosphate-buffered saline (0.14 mM NaCl, 2.7 mM KCl, 1.5 mM KH_2_PO_4_, 8.1 mM Na_2_HPO_4_; pH 7.0), and obtained samples were subjected within 30 min to the flow cytometry analysis on BD LSRFortessa SORP Cell Analyzer (BD Biosciences, Germany) using 488 nm laser. Before measurements, the probes were vigorously vortexed to disrupt cell clumps formed in bacterial suspensions left without shaking. 50,000 individual cells were analyzed in each experimental run. Recorded events were further gated to exclude cell duplets and debris using forward scatter (FSC) and side scatter (SSC) plots (SSC-H vs SSC-W and FSC-A vs SSC-A). Data were analyzed using FlowJo software version v10.7.1 (FlowJo LLC, Ashland, OR, USA). The gate used to differentiate subpopulation of negative cells from curli-positive was defined based on W3110 RpoS^+^ strain carrying no fluorescent *csgBA* reporter.

### Statistical analysis

Expression of *csgBA* genes and fraction of curli-positive cells were statistically analyzed using ordinary one-way analysis of variance followed by Dunnett’s test to run multiple comparisons between WT and deletion strains. The significance level was set to 0.05. The statistical analysis was performed in GraphPad Prism version 10.0.0 for Windows (GraphPad Software, Boston, Massachusetts USA).

## RESULTS

### Multiple regulators control expression of curli fibers in planktonic culture

In order to systematically investigate the role of different cellular factors in the expression of curli structural genes in *E. coli*, we utilized a recently described strain that encodes a chromosomal GFP reporter as a part of the *csgBA* operon (45, 47) (Fig. S1). This transcriptional reporter does not interfere with biofilm formation, and it was previously used to investigate the *csgBA* expression in both planktonic and biofilm cultures (45, 47). We first constructed a library of 32 mutants in this strain background, each carrying a deletion of a single gene selected based on its known involvement in extracellular matrix formation, control of cell stress response, metabolic transitions, and maintenance of DNA architecture or cross-regulation between motility and curli production.

**Fig 1:**
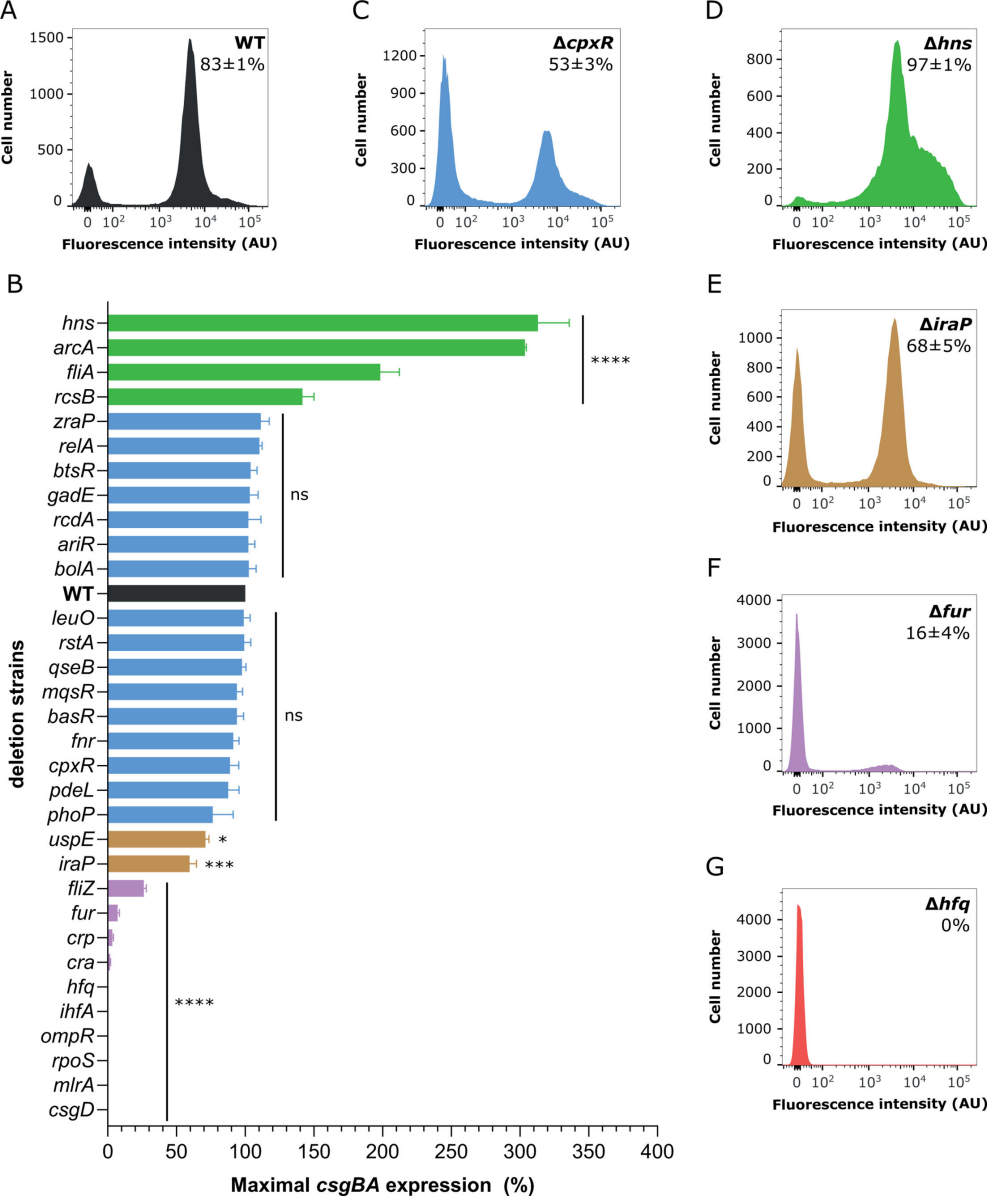
Effects of regulator gene deletions on curli gene expression. *E. coli* cells carrying genomic sfGFP transcriptional reporter of the *csgBA* operon were grown in flasks with TB at 30°C under constant shaking for a time required to reach maximum levels of the *csgBA* reporter activity (17–27 h; see Materials and Methods) and then subjected to the flow cytometry analysis. (**A**) Distribution of single-cell fluorescence levels in the WT cell population. (**B**) Activity of the *csgBA* transcriptional reporter in WT (black) and indicated gene deletion strains with either enhanced (green), unaffected (blue), reduced (brown), strongly impaired (purple), and entirely abolished (red) curli gene expression. Error bars indicate standard error of the mean of at least three biological replicates. Not significant (ns) at *P* > 0.01, * at *P* = 0.01–0.05, *** at *P* = 0.001–0.0001, **** at *P* < 0.0001. (**C–G**) Distributions of single-cell fluorescence levels in cell populations of indicated gene deletion strains with unaffected (**C**), enhanced (**D**), reduced (**E**), strongly impaired (**F**), and entirely abolished (**G**) *csgBA* expression. Percentage of curli-positive cells in the population (mean of at least three biological replicates ±SD) is indicated for each strain. Note that the scale of the *Y*-axes differs between strains to improve readability.

We next measured activity of the *csgBA* transcriptional reporter in planktonic cultures of these mutants, grown with vigorous shaking to avoid cell aggregation (47), using flow cytometry. In order to account for possible growth differences between strains, we monitored reporter expression over time and used maximal expression levels for this analysis. Consistent with previous findings, expression of the *csgBA* genes was bimodal, with a fraction of cells (~80%) activating GFP reporter in the overnight culture of the WT strain background (Fig. 1A) (47). Half of the obtained deletion mutants showed the *csgBA* expression similar to that of WT (Fig. 1B and C and Fig. S2). Notably, these also included genes encoding regulators that were previously suggested by *in vivo* or *in vitro* experiments to bind to the *csg* intergenic region and/or modulate curli expression, either positively (*basR*, *bolA*, *rcdA*, and *rstA*) (16, 59–61) or negatively (*btsR*, *mqsR*, *cpxR*, and *rstA*) (16, 33, 35, 36, 62–64). Similarly, deletion of *ariR*, *fnr*, *phoP*, and *gadE* had no impact on the average *csgBA* expression levels (Fig. 1B and Fig. S2), although they led to moderate changes in the numbers of curli-expressing cells (Fig. S3).

Four mutants (Δ*hns*, Δ*arcA*, Δ*fliA*, and Δ*rcsB*) showed increased activity of the *csgBA* reporter as well as the number of curli-positive cells, with the effects of *hns* and *arcA* deletions being most pronounced (Fig. 1B and D; Fig. S3 and S4A). Moderate reduction of the *csgBA* expression and the number of curli-expressing cells was observed in mutants lacking genes that encode stress response regulators UspE and IraP*,* indicating positive but auxiliary involvement of these proteins in the regulation of expression of curli structural genes in a well-shaken *E. coli* planktonic culture (Fig. 1B and E; Fig. S3 and S4B). In contrast to the positive impact of *fliA* deletion, deletion of *fliZ* showed decreased activity of the *csgBA* reporter. Deletion of genes encoding global regulators of carbon metabolism *cra* and *crp* and the regulator of iron homeostasis *fur* severely reduced expression of the *csgBA* operon (Fig. 1B and F; Fig. S3 and S4C).

Finally, the expression of *csgBA* genes was completely abolished by the removal of regulators known to directly bind to the *csg* intergenic region and be required for the activation of curli gene expression (*rpoS*, *mlrA*, *csgD*, *ompR*, and *ihfA*) (Fig. 1B; Fig. S3 and S4D) (8). Similar abolishment was observed upon disruption of *hfq* gene encoding a chaperone for sRNAs (Fig. 1B and G; Fig. S3). Since both, the activator of curli gene expression IHF and the inhibitor H-NS, have chromosome-organizing function, we additionally created a strain lacking both respective genes. This double-deletion strain showed only weakly positive and monomodal expression of the *csgBA* operon (Fig. S4E), suggesting dominance of the activatory effect of IHF and the importance of the interplay between the two factors for the establishment of bimodality of curli gene expression.

### Effects of regulator gene deletions on cell growth and dynamics of *csgBA* reporter activation

**Fig 2:**
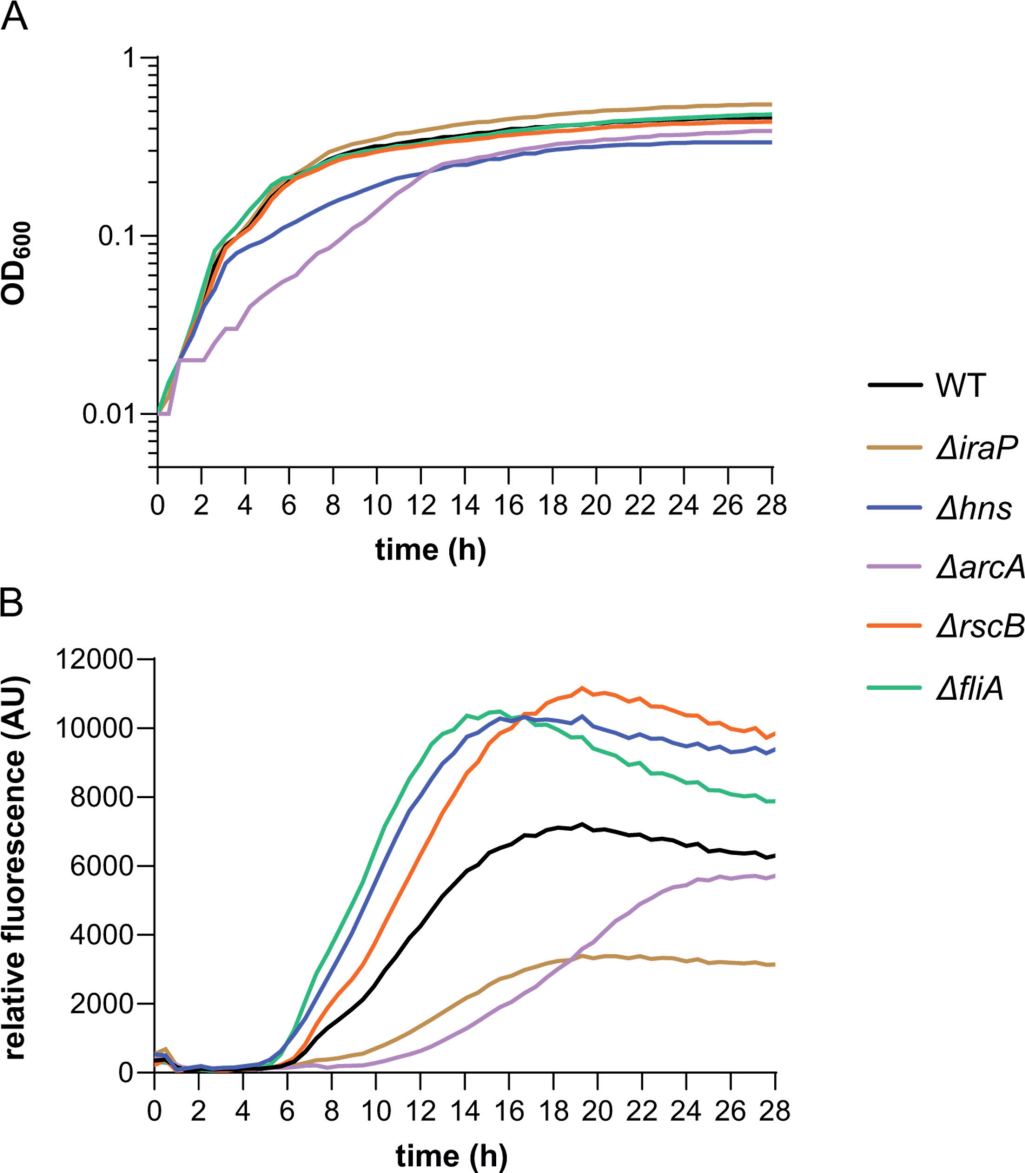
Effects of regulator gene deletions on curli activation pattern. *E. coli* cells carrying genomic sfGFP transcriptional reporter of the *csgBA* operon were grown in a plate reader in TB at 30°C with shaking. Optical density at 600 nm (OD_600_) (**A**) and relative fluorescence (absolute fluorescence/OD_600_) (**B**) for WT and indicated gene deletion strains are shown. Graphs are representative of at least three independent replicates (other replicates are shown in Fig. S7).

In order to further explore how removal of individual regulators influences the dynamics of curli gene expression throughout the growth phase, we monitored cell growth and activity of the *csgBA* reporter over time using plate reader. The observed *csgBA* expression levels were generally consistent with those measured in the batch culture experiment using flow cytometry (Fig. 2; Fig. S5 through S7). An exception was the *arcA* mutant, which showed either the same as WT or weaker activation of curli fiber gene expression in the plate reader (Fig. 2B and Fig. S7B). This apparent difference was likely because of the slower growth of this mutant compared to WT (Fig. 2A; Fig. S7A), which therefore reaches maximal level of curli expression only at a later time point (25-30 h for Δ*arcA* compared to 22-25 h for WT) in cultures grown in flasks for flow cytometry measurements. Nonetheless, the deletion of *arcA* and even more so of *hns* led to activation of *csgBA* expression at lower cell density, consistent with derepression of curli genes in these deletion strains. In contrast, removal of IraP—positive regulator of RpoS—delayed activation of curli gene expression (Fig. 2 and Fig. S7). These observations were further confirmed by using flow cytometry to monitor over time reporter expression in corresponding strains grown in flasks (Fig. S8). Also in these measurements, curli gene expression in the Δ*hns* strain was heterogeneous but not as clearly bimodal as in other strains.

### Complementation of curli-deficient strains by ectopic expression of *csgD*

**Fig 3:**
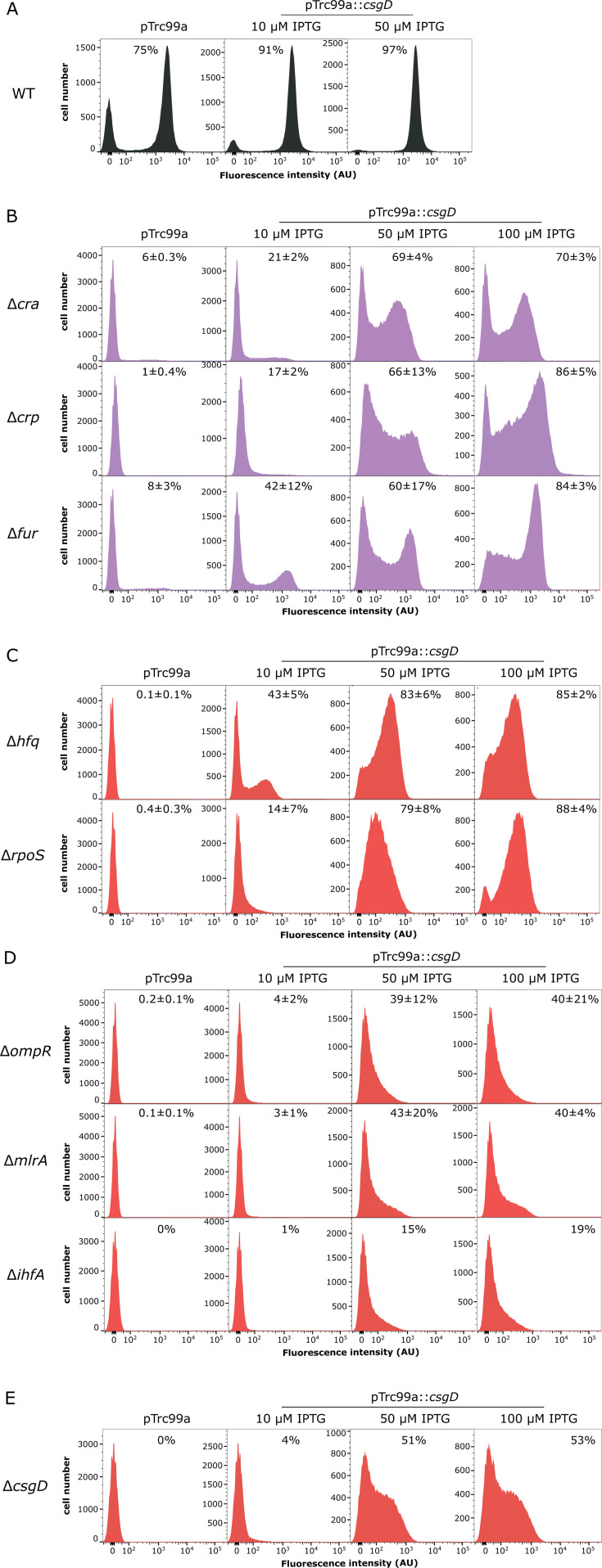
Strain-dependent restoration of the *csgBA* expression by ectopic production of CsgD. *E. coli* WT cells carrying genomic sfGFP transcriptional reporter of the *csgBA* operon (**A**) and the indicated gene deletion strains with strongly impaired (**B**) or abolished (**C–E**) reporter expression were transformed with either empty pTrc99a plasmid (control) or with pTrc99a plasmid carrying *csgD* gene (pTrc99a::*csgD*). Bacterial cultures were grown and subjected to the flow cytometry analysis as in Fig. 1. Expression was induced with 10, 50, or 100 µM IPTG, as indicated; control plasmid was induced with 100 µM IPTG to rule out possible unspecific effects of induction. Colors correspond to Fig. 1. Distributions of single-cell fluorescence levels are shown. Percentage of curli-positive cells in the population is indicated for each strain (mean of at least three biological replicates ±SD; except for Δ*ihf* and Δ*cgsD* strains where only one biological replicate was quantified). Note that the scale of the *Y*-axes differs between individual strains to improve readability.

We further investigated whether the negative impact of gene deletions on the *csgBA* expression could be rescued by production of their immediate upstream regulator CsgD from a plasmid vector under control of the IPTG-inducible promoter. As expected, the expression of *csgD* at higher induction levels resulted in activation of curli gene reporter in almost all WT cells (Fig. 3A). Restoration of the *csgBA* expression was observed in *fur*, *crp*, *cra*, and *hfq* deletion strains, with formation of a well-distinguishable subpopulation of curli-positive cells at intermediate induction levels (Fig. 3B). Our results thus suggest that these four regulators are primarily required for regulation upstream of *csgD* and are not essential for bimodal expression of curli fibers, since the bimodality of the *csgBA* expression could be restored by the expression of CsgD from the inducible plasmid vector that is likely monomodal. Ectopic expression of *csgD* in the Δ*hfq* background also resulted in the increased number of curli-positive cells, but with lower reporter expression levels in positive cells compared to WT, and hence lesser separation between curli-positive and curli-negative cells (Fig. 3C). CsgD could further activate *csgBA* expression in the *rpoS* deletion strain (Fig. 3C), which confirms that the expression of curli structural genes can be induced from a vegetative promoter (12, 20, 32, 65–67). Reduced separation between curli-positive and curli-negative cell subpopulations in the Δ*hfq* and Δ*rpoS* strains indicates that the RpoS- and sRNA-dependent regulation contributes to the bimodality of curli gene expression.

In contrast, strains carrying deletions of *ihfA*, *ompR*, or *mlrA* genes demonstrated only partial recovery of curli expression, without formation of a distinct positive population even at high IPTG induction levels (Fig. 3D). This suggests that their gene products are involved in the regulation of curli gene expression downstream of CsgD and might modulate bimodal activation of the *csgBA* expression. Notably, although complementation by CsgD produced from the plasmid vector was better in the *csgD* deletion strain (lacking 421 nucleotides of the coding sequence [see Materials and Methods for details]) compared to Δ*ihfA*, Δ*ompR*, or Δ*mlrA* mutants, it failed to fully restore the *csgBA* expression, indicating that the native regulation of *csgD* and/or gene regulatory elements within its coding sequence might be required to fully induce expression of curli structural genes.

### Deletions of several DGC genes affect curli gene expression

**Fig 4:**
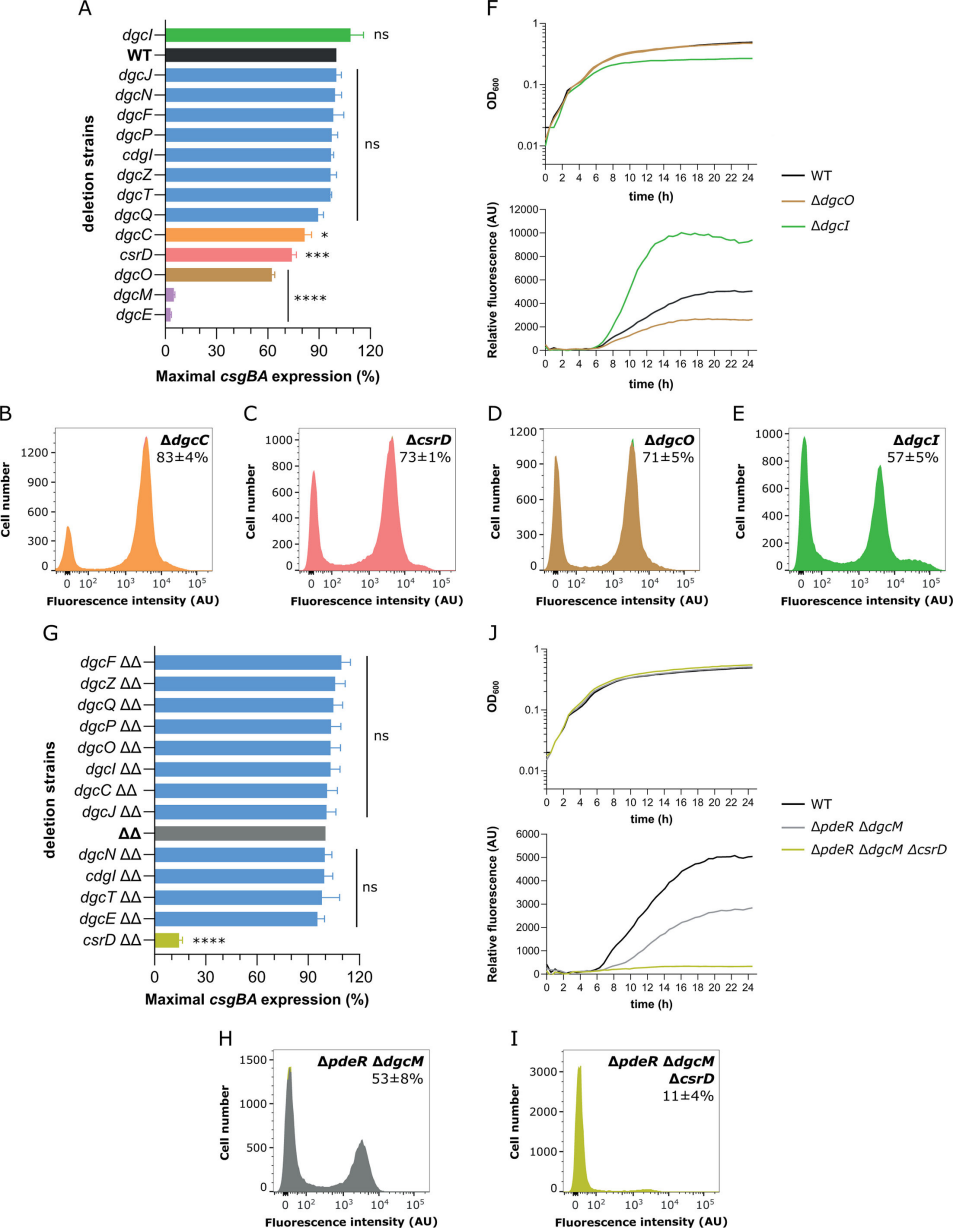
Regulation of curli expression by DGCs dependent on the DgcM/PdeR module. Activity of the *csgBA* transcriptional reporter (**A, G**) and distribution of single-cell fluorescence levels (**B–E, H, I**) in strains lacking individual DGCs. Gene deletions were introduced either in the WT background (**A–E**) or in the Δ*dgcM* Δ*pdeR* background (indicated by ΔΔ) (**G–I**). WT is shown in black color, strain lacking the DgcM/PdeR regulatory module, in gray, and indicated gene deletion strains with unaffected curli expression, in blue, with affected, in different colors corresponding to the sign and magnitude of the effect. Error bars indicate standard error of the mean of at least three biological replicates. Not significant (ns) at *P* > 0.01, * at *P* = 0.01–0.05, *** at *P* = 0.001–0.0001, **** at *P* < 0.0001. *E. coli* cultures were grown in flasks for a time required to reach maximum levels of the *csgBA* reporter activity (19–28 h for WT and other strains, except 12–17 h for Δ*dgcI*) and then subjected to the flow cytometry analysis as in Fig. 1. Percentage of curli-positive cells in the population (mean of at least three biological replicates ±SD) is indicated for each strain. Note that the scale of the *Y*-axes differs between individual strains to improve readability. (**F, J**) Optical density (OD_600_) and relative fluorescence (absolute fluorescence/OD_600_) of WT and mutant strains lacking indicated DGCs during growth in a plate reader as in Fig. 2 (other replicates are shown in Fig. S13 through S16).

We next investigated how disruption of individual DGC-encoding genes affects *csgBA* expression in planktonic *E. coli* culture. Consistent with our previous study (47), deletions of the known curli regulators *dgcE* and *dgcM* nearly abolished curli gene expression (Fig. 4A and Fig. S9). Although removal of the majority of DGCs had no pronounced effects on the *csgBA* expression (Fig. 4A and Fig. S10A, S11A, S12), deletions of *dgcO*, *csrD*, or *dgcC* genes resulted in markedly lower activity of the *csgBA* reporter (Fig. 4A through D and Fig. S10A, S13). Thus, only these DGCs contribute to curli regulation in *E. coli* in addition to DgcE and DgcM under conditions used in this study. Deletion of *dgcI* gene had a more complex effect, leading to a decrease in the number of curli-expressing cells (Fig. S10A), as could be expected from the removal of a DGC, but also resulting in a fraction of curli-positive cells with highly elevated expression (Fig. 4E), and to transiently higher average expression level of curli reporter in cell population in the early growth phase (Fig. 4F and Fig. S13).

Since c-di-GMP is known to influence expression of the *csgBA* operon through the DgcM/PdeR-dependent *csgD* activation, we further measured reporter activity in triple mutants lacking DgcM/PdeR module and one of the individual DGCs (Fig. 4G). In agreement with our previous findings, activity of the *csgBA* operon was lower in a mutant lacking DgcM/PdeR regulatory module compared to WT but similarly bimodal (Fig. 4H and J; Fig. S14) (47). Consistent with the expected dependence of c-di-GMP regulation on the DgcM/PdeR module, deletions of other cyclase genes had no effect in this background (Fig. 4G and Fig. S10B, S11B, S15), including the deletions of *dgcO*, *dgcI*, and *dgcC*. In contrast, the deletion of the *csrD* gene encoding a degenerate DGC led to a much stronger decrease of the reporter activity in the *dgcM/pdeR* deletion background compared to the WT background (Fig. 4A, C, G and I; Fig. S10B and S16).

## DISCUSSION

*E. coli* and other enterobacteria are known to produce curli fibers in response to various environmental stresses. Multiple cellular factors were described to affect curli gene expression in biofilms, but the interpretation of previous studies was often complicated by environmental gradients in such structured communities. In this study, we systematically investigated the role of multiple cellular factors in regulation of curli gene expression under simpler conditions, in *E. coli* planktonic culture grown aerobically under continuous mixing.

Out of 32 tested strains deleted for individual transcription factors and stress response regulators, 21 were found to modulate the *csgBA* expression levels and/or the number of curli-expressing cells in our experiments (Fig. 1B). Curli expression was elevated in several deletion strains, including those lacking components of the Rcs and ArcA/B signaling systems. The repression of curli gene expression by these pathways is in good agreement with previous studies, with RcsB being known to negatively regulate *csgD* expression (26, 30, 68–71) and ArcA known to repress transcription of *rpoS* (22, 71, 72). Notably, in the latter case, reaching maximal curli expression required longer culture incubation because of the reduced growth rate of the Δ*arcA* strain. An even more dramatic enhancement of curli gene expression was observed upon the deletion of *hns* (Fig. 1B), confirming the importance of this DNA-organizing factor for repression of curli genes in *E. coli* (32). Interestingly, H-NS was shown to activate curli gene expression in *Salmonella* but to repress it in *E. coli*, by directly binding to the *csg* intergenic region and also by negatively affecting stability of the *rpoS* mRNA (16, 24, 66, 73, 74).

In contrast, two stress regulators, the universal stress factor UspE and the RpoS-stabilizing factor IraP (75–78), showed positive impact on curli gene expression. The effect of IraP is likely mediated by the modulation of RpoS stability (75), which is consistent with the delayed activation of the *csgBA* gene expression in the Δ*iraP* background. Our data further suggests the involvement of the iron-binding global transcriptional regulator Fur in positive regulation of curli genes, which may be related to the previously observed inhibition of curli gene expression and biofilm formation by iron chelation (79).

We further observed opposite effects of *fliZ* and *fliA* gene deletions, with curli gene expression being elevated in the Δ*fliA* strain but lowered in the Δ*fliZ* strain. The former effect was expected, and it could be explained by the FliA-dependent activation of expression of *pdeH*, the major PDE of *E. coli* (80) that inhibits curli gene expression by lowering global levels of c-di-GMP (81). However, FliZ was previously shown to antagonize the activity of RpoS by binding within RpoS-dependent promoters (37, 80). Why under our conditions, FliZ has a positive rather than negative impact on curli expression remains to be investigated, but it might be due to its interplay with other regulators.

Dramatic reduction of curli expression was observed in the absence of the global transcription factors Cra and CRP that are activated at low-nutrient conditions (11), thus confirming their importance for initiation of curli expression (8, 82–85). Similar abortion of curli expression was observed upon deletion of *hfq*. Hfq is a chaperone that promotes pairing between sRNAs and their target mRNA, thus helping to regulate mRNA stability in response to different types of cell stress (86, 87). The impact of *hfq* on curli gene expression is likely mediated by the altered activity of sRNAs that are known to modulate curli expression, either directly by binding to the *csg* intergenic region (25, 26, 30, 70, 88, 89) or indirectly, through regulation of other cell factors that affect curli production, including RpoS (90–92). Consistent with our observation, it has been previously shown that deletion of *hfq* leads to decreased *csgD* transcript levels in *S. enterica* (93). Importantly, the defect of curli expression in all of *crp*, *cra*, and *fur* deletion strains could be complemented by the production of CsgD from the inducible plasmid vector (Fig. 3B), confirming that these regulators act upstream of CsgD and are not required for the establishment of bimodality. In contrast, the complementation was only partial in the case of the *hfq* strain (Fig. 3C). Similarly, although the ectopic CsgD production restored expression of the *csgBA* genes in the absence of *rpoS*, the maximal level of expression was also lower. This indicated that a CsgD-independent impact of sRNA regulation on *csgBA* reporter activity might be mediated by RpoS. Furthermore, this data demonstrated that expression of curli structural genes can occur independently of RpoS, which is consistent with existence of RpoD-dependent promoter upstream of the *csgBAC* operon (12, 20, 32, 65–67). Another similarity between the *rpoS* and *hfq* strains was much reduced bimodality of curli gene expression, indicating that it is at least partly RpoS-dependent.

The impact on bimodality was even more pronounced for *ompR*, *mlrA*, and *ihfA* genes, whose loss led to the complete abolishment of the *csgBA* activity and could not be well compensated by the ectopically produced CsgD (Fig. 3D). The corresponding gene products are known to regulate transcription of *csgD* and have multiple binding sites within the *csg* intergenic region (16, 23, 33, 66, 94). Our results indicate that they may also directly activate transcription of curli structural genes and that this direct regulation plays an important role in the establishment of *csgBA* bimodality, possibly by stabilizing the positive expression state. On the other hand, the negative expression state may be stabilized due to the activity of H-NS, which also has multiple binding sites within the *csg* region (16, 66). Indeed, the *csg* intergenic region has strong inherent curvature and high AT content (95), which aids binding H-NS and IHF. This could induce sharp DNA bending (16) and, in turn, affect promoter recognition by RpoS (96) and/or accessibility of binding sites for transcription factors (97). Indeed, it was shown that OmpR can bind together with IHF to positively regulate curli gene expression, whereas H-NS and IHF act in competition (16). Further experiments are required to elucidate how DNA curvature and the interplay between H-NS and IHF may determine the bimodality of curli gene expression, but IHF appears to be required for the bimodal activation of the *csgBA* expression even in the absence of H-NS.

A number of transcription factors did not show any involvement in the regulation of curli fibers expression. These included LeuO, RelA, PdeL, PhoP, ZraP, and QseB, but also factors that were previously described as putative curli regulators (BolA) (61) or proposed to bind to *csg* intergenic region and effect curli expression in either positive (BasR, RcdA) (59, 60), negative (BtsR, MqsR) (62, 64) or dual (RstA) (16) manner (Fig. 1B). Although this discrepancy might be due to higher complexity of curli regulation in structured communities or differences in growth conditions, our experiments make it clear that these factors are not essential for curli regulation.

In addition to transcriptional regulators, several DGCs also showed effects on curli gene expression in planktonic *E. coli* culture. Deletion of *dgcO* and *dgcC* resulted in decreased levels of the *csgBA* expression, dependent on the presence of the DgcM/PdeR regulatory module (Fig. 4A and G). A previous study indicated that DgcO interacts with PdeR (29), but the underlying regulatory mechanism of how DgcO influences the expression of curli structural genes remains to be understood. Deletion of another DGC gene, *dgcI*, had a more complex effect, reducing the number of curli-positive cells at the late stage of growth—as might be expected from a DGC gene deletion—but increasing the expression level of curli genes during entry into the stationary phase (Fig. 4F). This indicates that DgcI might act not only as a cyclase but also through a different regulatory mechanism.

Finally, we found that CsrD protein with degenerate DGC activity (40) has a positive effect on curli expression that is particularly pronounced in the absence of the DgcM/PdeR module (Fig. 4A and G). CsrD was reported to enable the RNase E-mediated degradation of sRNAs CsrB and CsrC (98, 99), which are known to suppress *csgD* expression (90). Moreover, interaction between CsrD and DgcM was previously observed *in vitro* (29), raising a possibility that CsrD might be sequestered by the DgcM/PdeR complex.

Besides, our work enables further insights into the bimodality of curli fiber production in *E. coli* cell population, although the underlying mechanism appears to be complex and multifactorial. Previous study has shown that *csgD* expression in planktonic culture is bimodal (or multimodal) and concluded that this bimodality emerges due to the regulation of *csgD* expression by the DgcM/PdeR complex (44) that might act as a bistable switch (100). However, this regulation cannot fully explain the observed bimodality of *csgBA* expression. Firstly, the deletion of *pdeR* along with its counterpart *dgcM* results in the bimodal expression of *csgBA* reporter comparable to those in the WT (47) (Fig. 1A and 4H). This demonstrates that the DgcM/PdeR-dependent regulation is not required for bimodality, although it may enhance robustness of bimodal differentiation (47). Secondly, the monomodal expression of *csgD* in Δ*csgD* background from an inducible vector leads to at least partly bimodal activation of *csgBA* reporter (Fig. 3E), suggesting existence of an additional mechanism that generates bimodality downstream of *csgD*. This mechanism appears to rely on the interplay between H-NS and IHF, which determine the DNA curvature of the *csg* intergenic region, and MlrA and OmpR, that may directly contribute to the activation of the *csgB* promoter besides their known activatory effect on the *csgD* promoter.

## Data Availability

All data needed to evaluate the conclusions are present in the article and its supplemental material.

## SUPPLEMENTAL MATERIAL

Supplemental figures and tables jb.00281-25-s0001.pdf Figures S1 to S16, and Tables S1 and S2.

ASM does not own the copyrights to Supplemental Material that may be linked to, or accessed through, an article. The authors have granted ASM a non-exclusive, world-wide license to publish the Supplemental Material files. Please contact the corresponding author directly for reuse.
