# Supplementary material for: Effects of multiple cell regulators on curli gene expression in Escherichia coli
Source: J Bacteriol. 2025 Nov 12;207(12):e00281-25. doi: 10.1128/jb.00281-25 (PMC12713406; doi:10.1128/jb.00281-25)
Supplement: Supplemental figures and tables — Figures S1 to S16, and Tables S1 and S2. [file jb.00281-25-s0001.pdf]

Supplemental Material

Effects of multiple cell regulators on curli gene expression in *Escherichia coli*

Maryia Ratnikava[1], Olga Lamprecht[1], Victor Sourjik[1]*

[1]Max Planck Institute for Terrestrial Microbiology and Center for Synthetic Microbiology (SYNMIKRO), Karl-von-Frisch Strasse 14, 35043 Marburg, Germany

*Corresponding author: Victor Sourjik, victor.sourjik@synmikro.mpi-marburg.mpg.de

Supplemental Figures

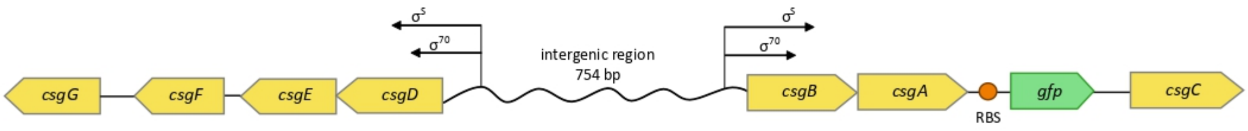

Figure S1. Schematic representation of the chromosomal transcriptional *csgBA* reporter. The *sfgfp* codon-optimized gene encoding superfolder green fluorescent protein (sfGFP) was cloned downstream of *csgA* with a strong ribosome binding site (RBS; 5'-ACAACTTAAGGAGGTATTC-3') as part of the same polycistronic RNA.

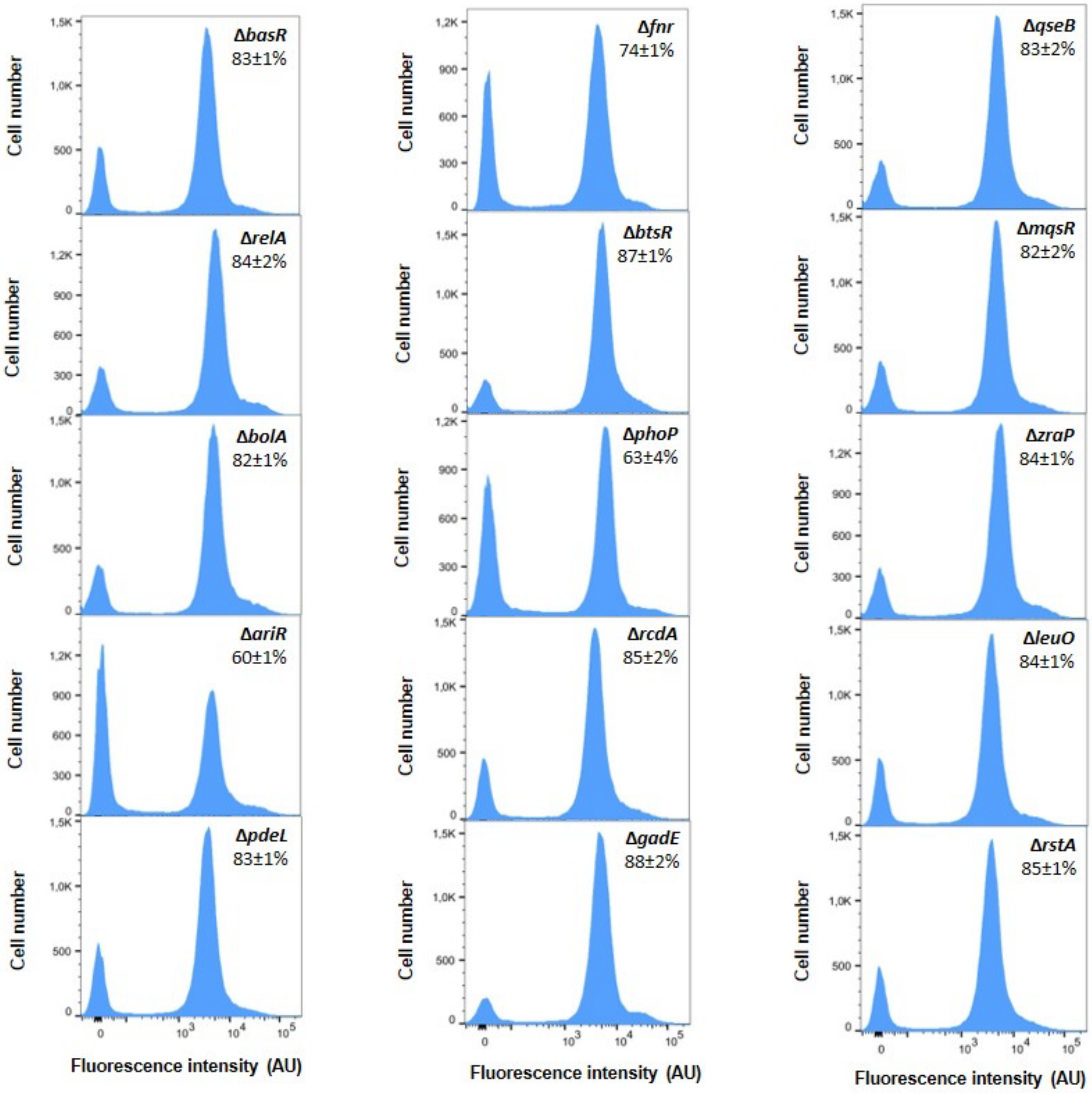

Figure S2. Gene deletions with no effect on curli gene expression. Data are for the same *E. coli* cultures as in Figure 1. Percentage of curli-positive cells in the population (mean of at least 3 biological replicates ± SD) is indicated for each strain. Note that the scale of the *y* axes differs between individual strains to improve readability.

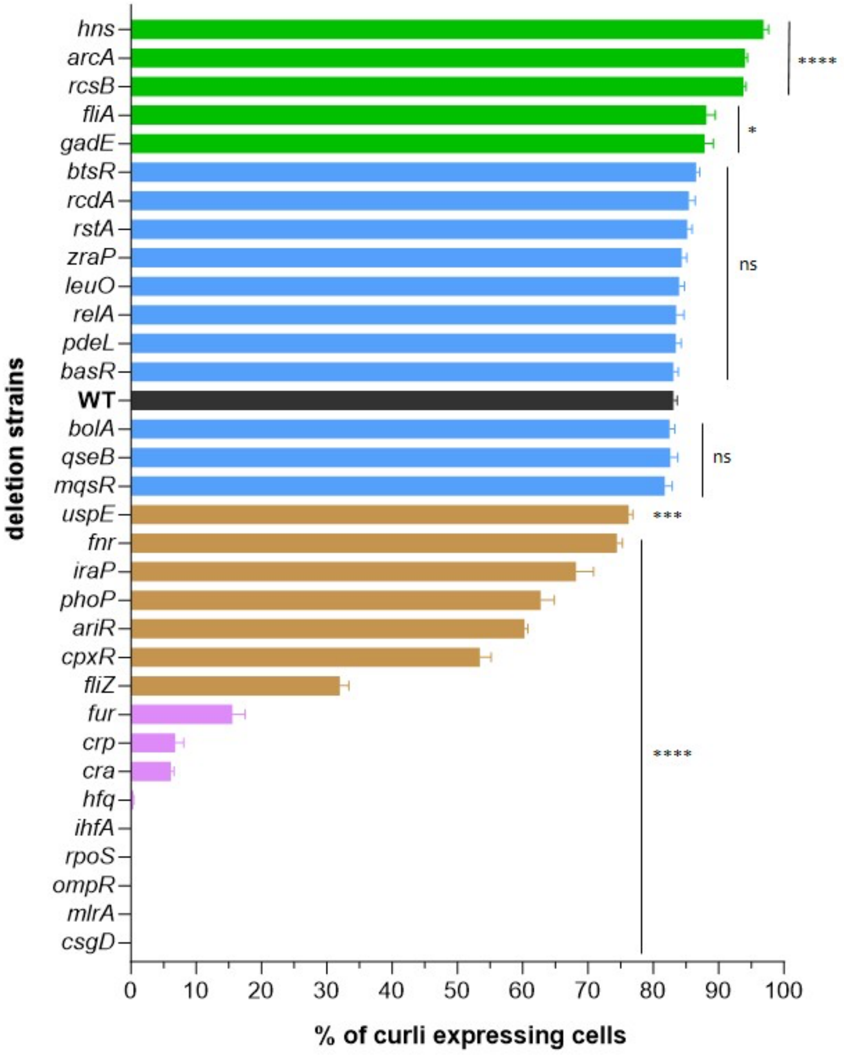

Figure S3. Percentage of curli-positive cells in indicated gene deletion mutants and WT. Data are for the same *E. coli* cultures as in Figure 1. WT is shown in black color; gene deletion strains with enhanced curli expression are indicated in green, with unaffected – in blue, reduced – in brown, strongly impaired – in purple and entirely abolished – in red. Error bars indicate SEM of at least 3 biological replicates. * at p = 0.01-0.05, ** at p = 0.01-0.001, *** at p = 0.001-0.0001, **** at p < 0.0001.

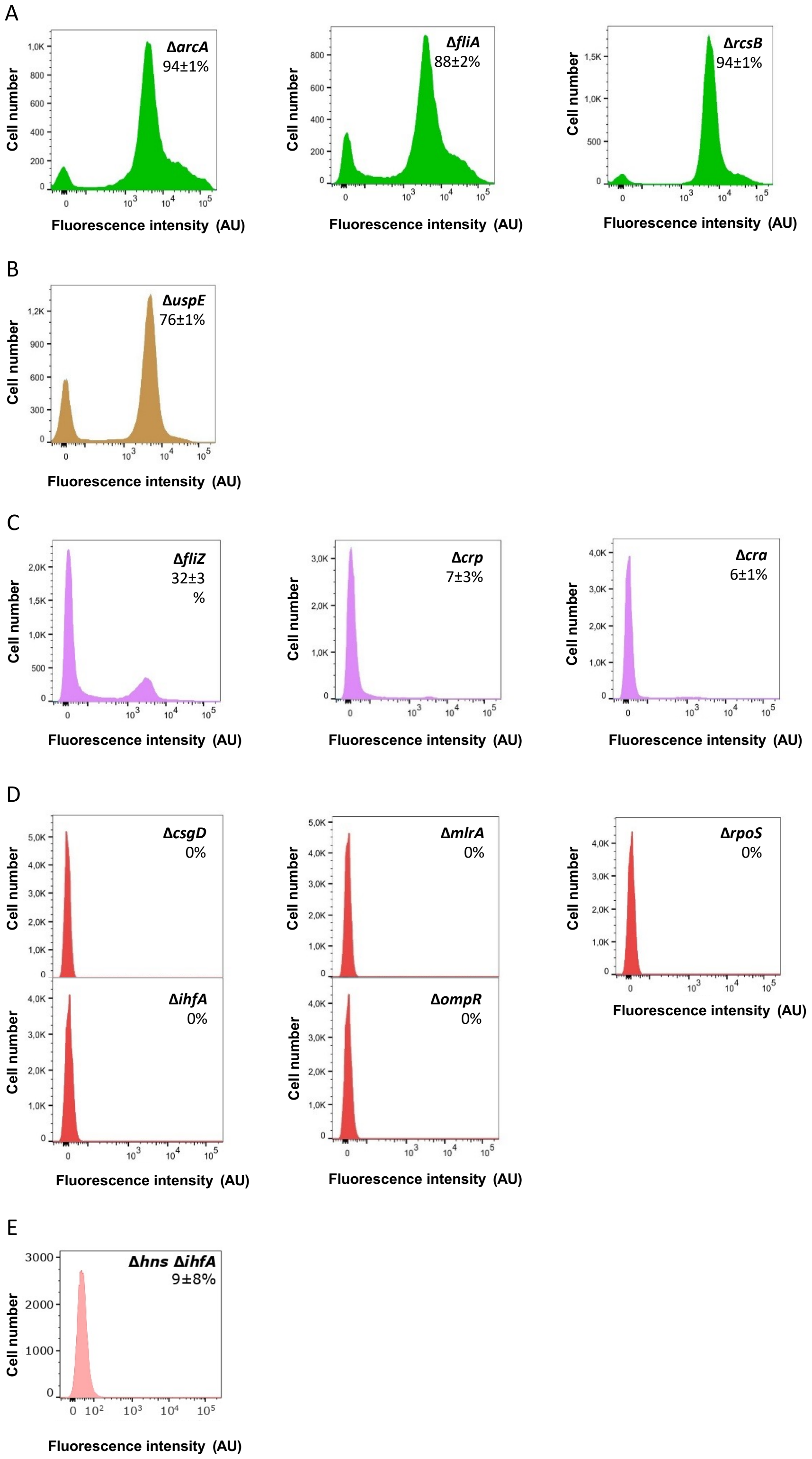

Figure S4. Gene deletions with impact on curli gene expression. **(A-C)** Distribution of single-cell fluorescence levels in individual gene deletion strains with enhanced **(A)**, moderately reduced

(B), strongly impaired **(C)** and entirely abolished **(D)** curli expression. Data are for the same *E. coli* cultures as in Figure 1. **(E)** Distribution of single-cell fluorescence levels in the Δ*hns* Δ*ihfA* strain, grown as in Figure 1. Percentage of positive cells in the population (mean of at least 3 biological replicates ± SD) is indicated for each strain. Note that the scale of the *y* axes differs between individual strains to improve readability.

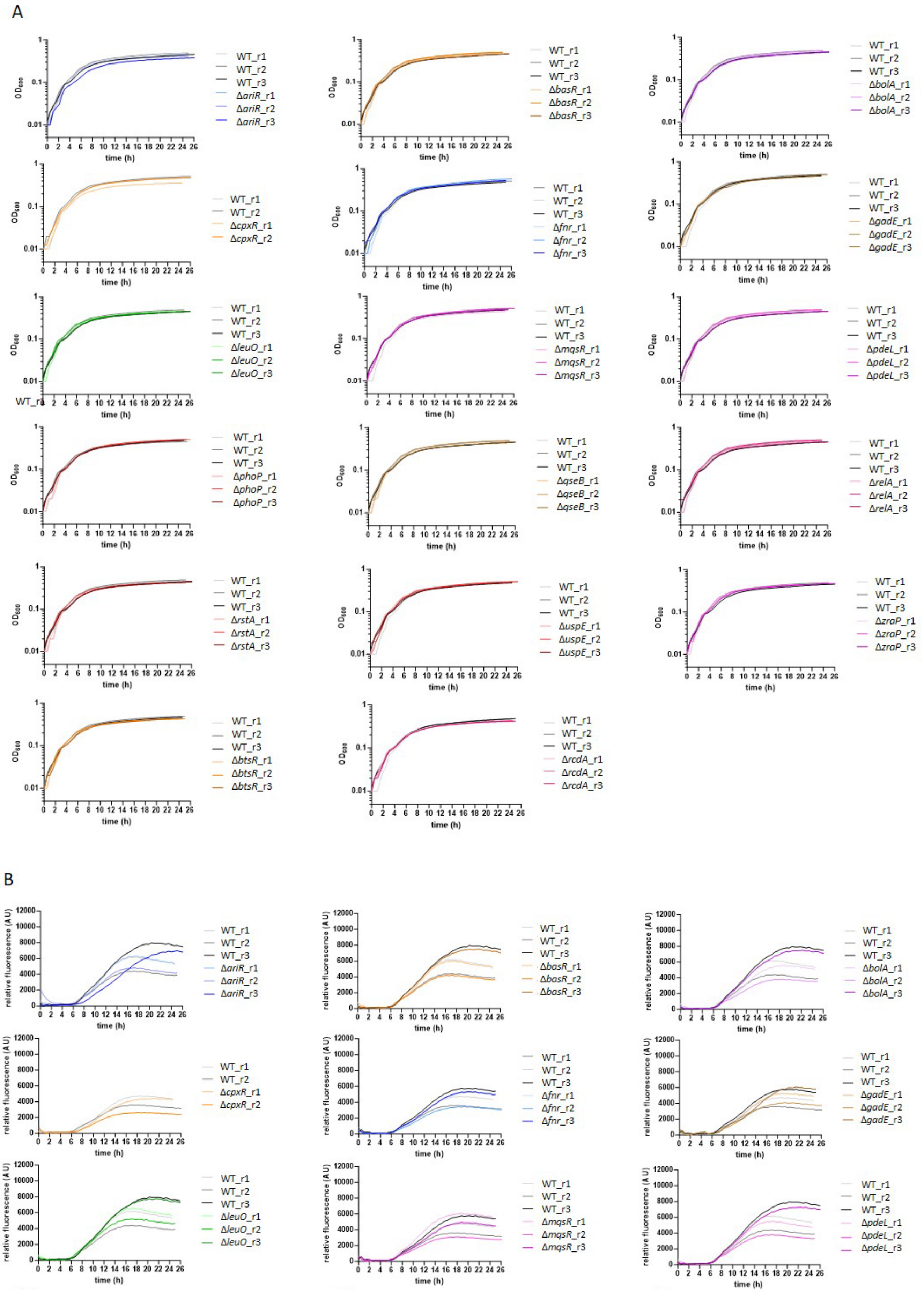
A
OD600
time (h)
WT_r1
WT_r2
WT_r3
ΔariR_r1
ΔariR_r2
ΔariR_r3
ΔbasR_r1
ΔbasR_r2
ΔbasR_r3
ΔbolA_r1
ΔbolA_r2
ΔbolA_r3
ΔcpxR_r1
ΔcpxR_r2
Δfnr_r1
Δfnr_r2
Δfnr_r3
ΔgadE_r1
ΔgadE_r2
ΔgadE_r3
ΔleuO_r1
ΔleuO_r2
ΔleuO_r3
ΔmqsR_r1
ΔmqsR_r2
ΔmqsR_r3
ΔpdeL_r1
ΔpdeL_r2
ΔpdeL_r3
ΔphoP_r1
ΔphoP_r2
ΔphoP_r3
ΔqseB_r1
ΔqseB_r2
ΔqseB_r3
ΔrelA_r1
ΔrelA_r2
ΔrelA_r3
ΔrstA_r1
ΔrstA_r2
ΔrstA_r3
ΔuspE_r1
ΔuspE_r2
ΔuspE_r3
ΔzraP_r1
ΔzraP_r2
ΔzraP_r3
ΔbtsR_r1
ΔbtsR_r2
ΔbtsR_r3
ΔrcdA_r1
ΔrcdA_r2
ΔrcdA_r3
B
relative fluorescence (A U)
time (h)

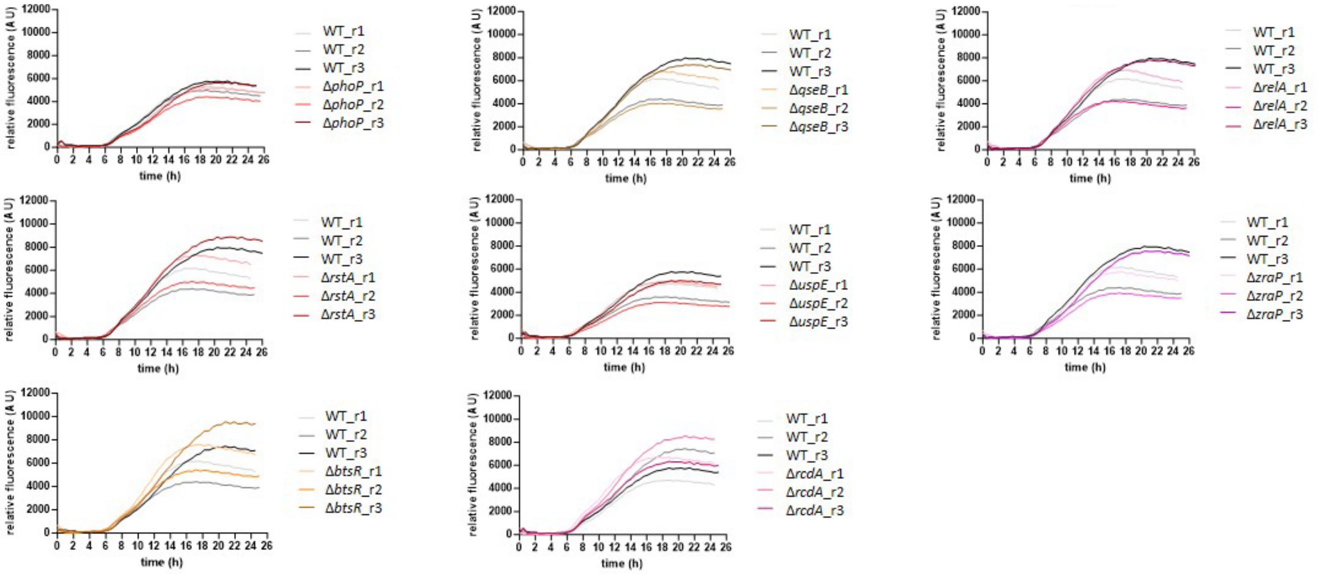

Figure S5. Gene deletions that do not alter curli gene expression throughout growth. *E. coli* cultures were grown in a plate reader as in Figure 2. **(A)** Optical density at 600 nm (OD_{600}) and **(B)** relative fluorescence (absolute fluorescence/OD_{600}) for WT and indicated gene deletion mutants are shown. r1-r3 refer to 3 independent replicates; in each replicate WT and the indicated deletion mutant were grown in the same experiment.

A

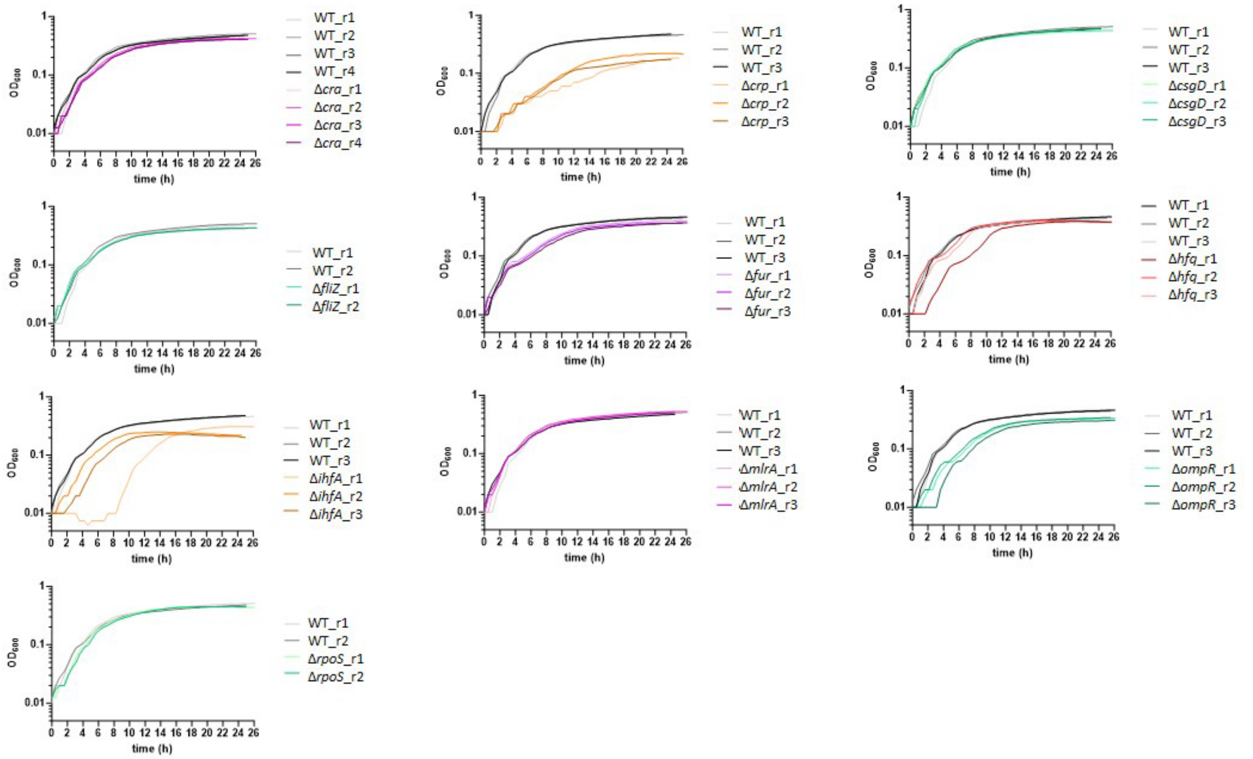
OD600
time (h)
WT_r1
WT_r2
WT_r3
WT_r4
Δcra_r1
Δcra_r2
Δcra_r3
Δcra_r4
Δcrp_r1
Δcrp_r2
Δcrp_r3
ΔcsgD_r1
ΔcsgD_r2
ΔcsgD_r3
ΔfliZ_r1
ΔfliZ_r2
Δfur_r1
Δfur_r2
Δfur_r3
Δhfq_r1
Δhfq_r2
Δhfq_r3
ΔihfA_r1
ΔihfA_r2
ΔihfA_r3
ΔmlrA_r1
ΔmlrA_r2
ΔmlrA_r3
ΔompR_r1
ΔompR_r2
ΔompR_r3
ΔrpoS_r1
ΔrpoS_r2

B

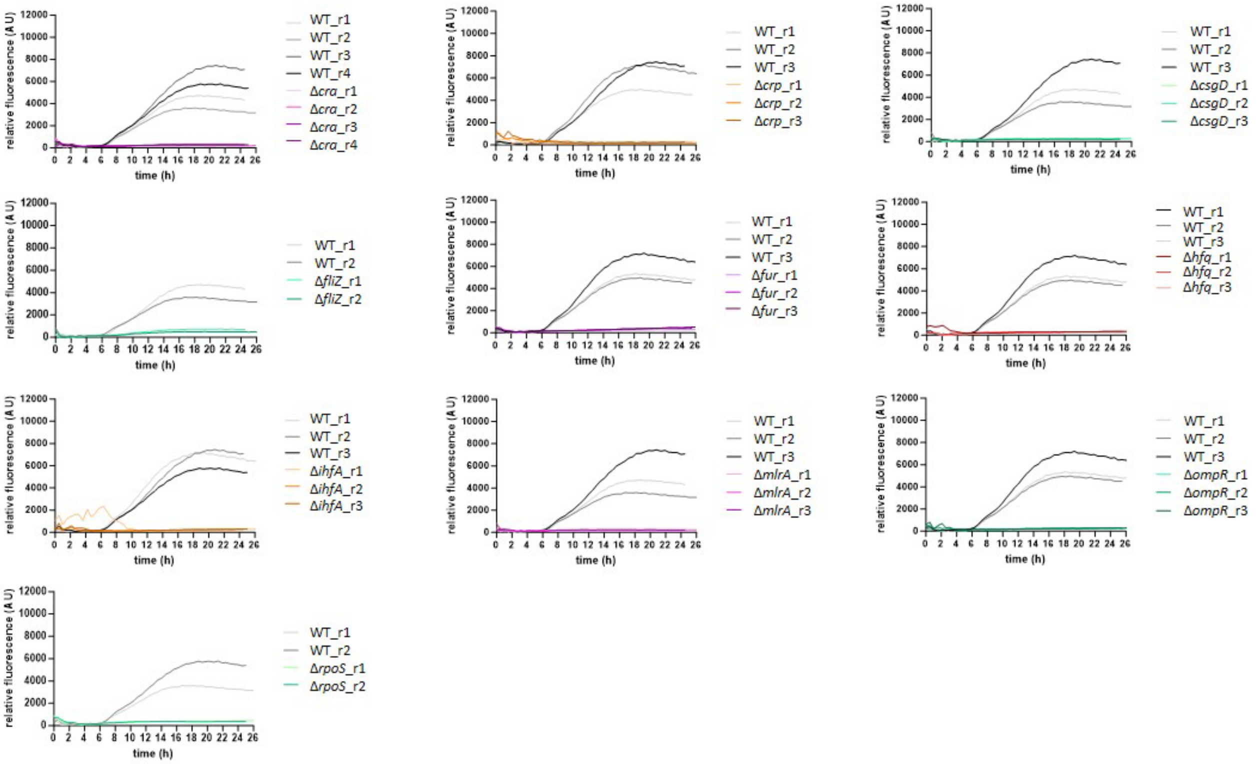
relative fluorescence (A U)
time (h)
WT_r1
WT_r2
WT_r3
WT_r4
Δcra_r1
Δcra_r2
Δcra_r3
Δcra_r4
Δcrp_r1
Δcrp_r2
Δcrp_r3
ΔcsgD_r1
ΔcsgD_r2
ΔcsgD_r3
ΔfliZ_r1
ΔfliZ_r2
Δfur_r1
Δfur_r2
Δfur_r3
Δhfq_r1
Δhfq_r2
Δhfq_r3
ΔihfA_r1
ΔihfA_r2
ΔihfA_r3
ΔmlrA_r1
ΔmlrA_r2
ΔmlrA_r3
ΔompR_r1
ΔompR_r2
ΔompR_r3
ΔrpoS_r1
ΔrpoS_r2

Figure S6. Gene deletions that inhibit curli gene expression throughout growth. *E. coli* cultures were grown in a plate reader as in Figure 2. **(A)** Optical density at 600 nm (OD_{600}) and **(B)** relative fluorescence (fluorescence/OD_{600}) for WT and indicated deletion mutants are shown. r1-r3 refer to 3 independent replicates; in each replicate WT and the indicated gene deletion mutant were grown in the same experiment.

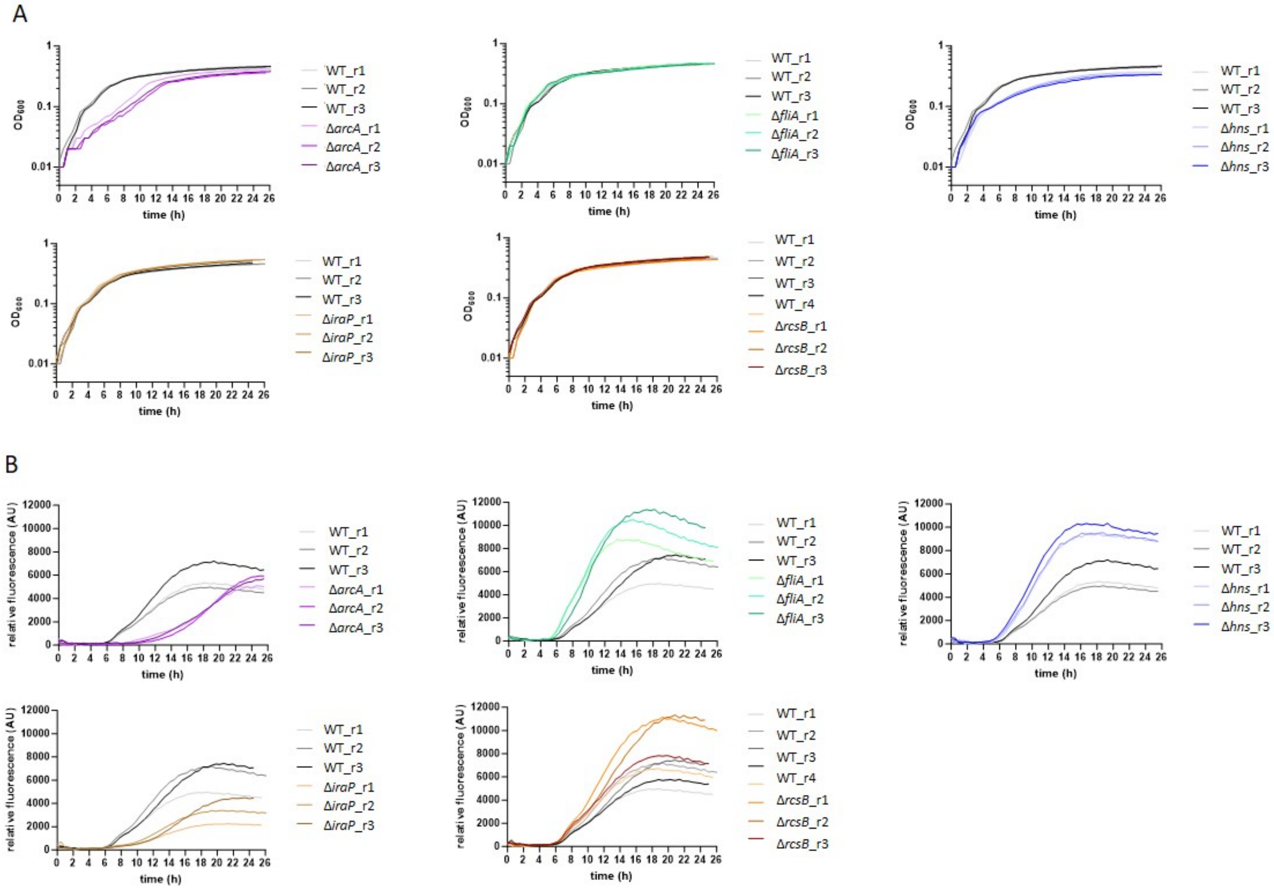

Figure S7. Gene deletions that alter curli gene expression throughout growth. *E. coli* cultures were grown in a plate reader as in Figure 2. **(A)** Optical density at 600 nm (OD_{600}) and **(B)** relative fluorescence (absolute fluorescence/OD_{600}) for WT and indicated deletion mutants are shown. r1-r3 refer to 3 independent replicates; in each replicate WT and the indicated gene deletion mutant were grown in the same experiment.

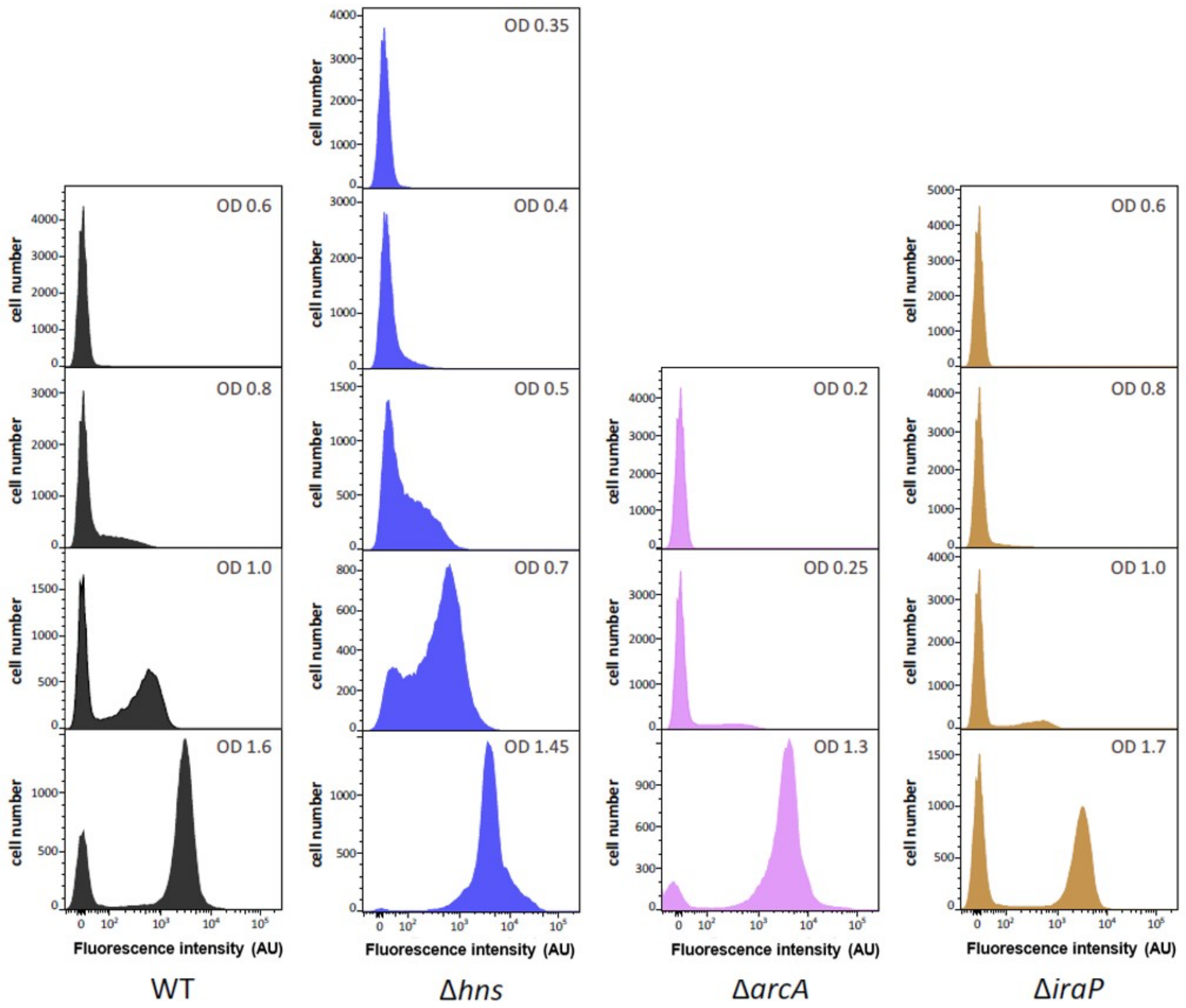

Figure S8. Time-dependence of the *csgBA* operon expression in WT and indicated gene deletion strains in flasks.

E. coli cells carrying genomic transcriptional reporter of the *csgBA* operon were grown in flasks and subjected to the flow cytometry analysis as in Figure 1. Distribution of single-cell fluorescence levels in individual gene deletion strains is shown. Note that the scale of the *y* axes differs between individual strains to improve readability.

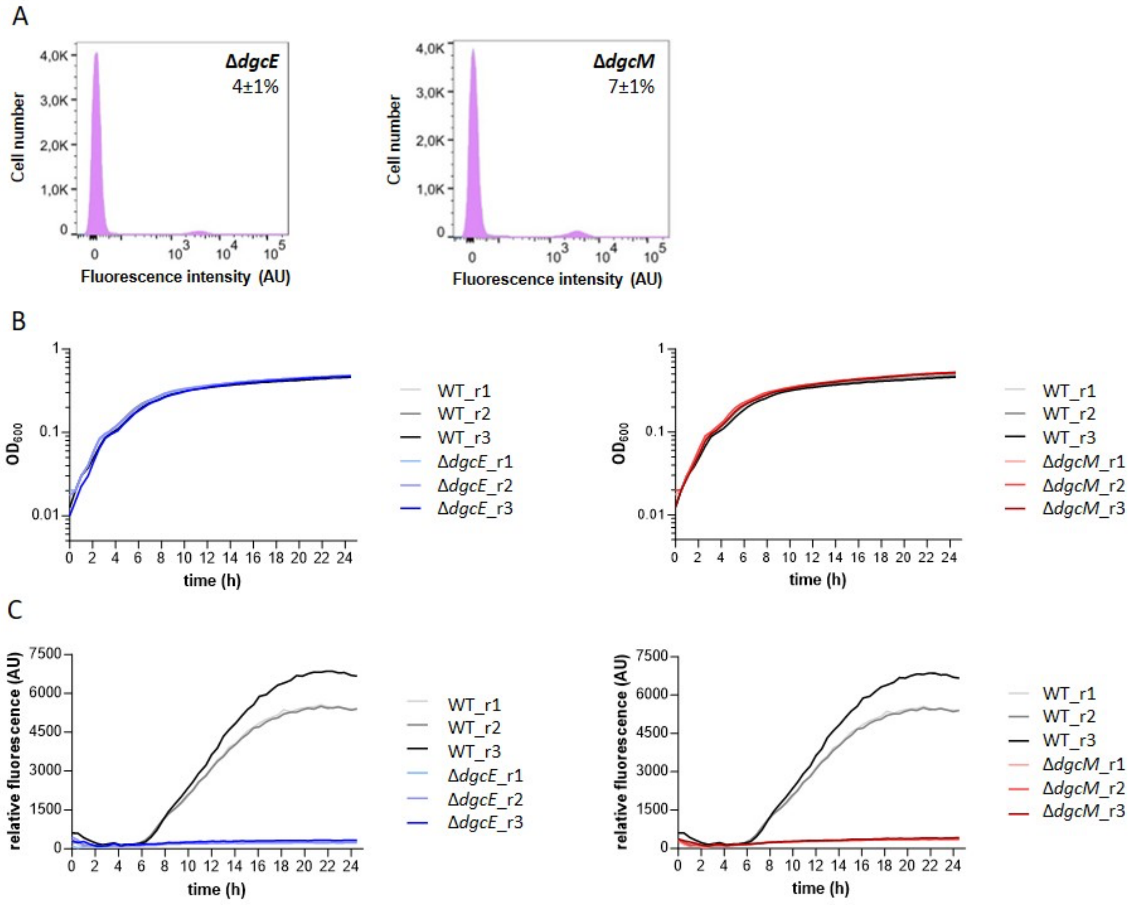

Figure S9. Deletion of *dgcE* or *dgcM* disrupts curli gene expression. (A) Distribution of single-cell fluorescence levels in deletion strains lacking *dgcE* or *dgcM*. *E. coli* cells were grown in flasks and subjected to the flow cytometry analysis as in Figure 1. Fraction of positive cells in the population (mean of at least 3 biological replicates ± SD) is indicated for each strain. r1-r3 refer to 3 independent replicates; in each replicate WT and the indicated deletion mutant were grown in the same experiment. **(B)** Optical density (OD_{600}) and **(C)** relative fluorescence (absolute fluorescence/OD_{600}) are shown for WT and mutant strains lacking indicated diguanylate cyclases during growth in a plate reader as in Figure 2.

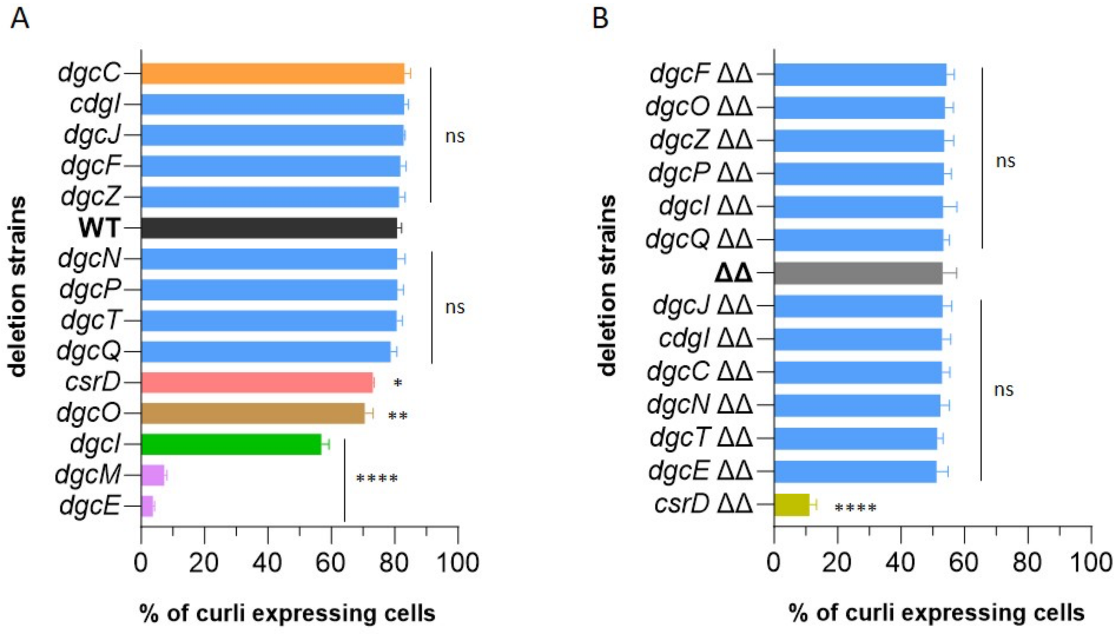

Figure S10. Percentage of curli-expressing cells in mutants with disrupted *dgc* genes. Gene deletions were introduced either in the WT background **(A)** or in the Δ*dgcM* Δ*pdeR* background (indicated by ΔΔ) **(B)**. Data are for the same *E. coli* cultures as in Figure 4. WT is shown in black color and deletion strains with unaffected curli expression – in blue, with affected – in different colors (color code is same as in Figure 4). Error bars indicate SEM of at least 3 biological replicates. * at p = 0.01-0.05, ** at p = 0.01-0.001, *** at p = 0.001-0.0001, **** at p < 0.0001

A

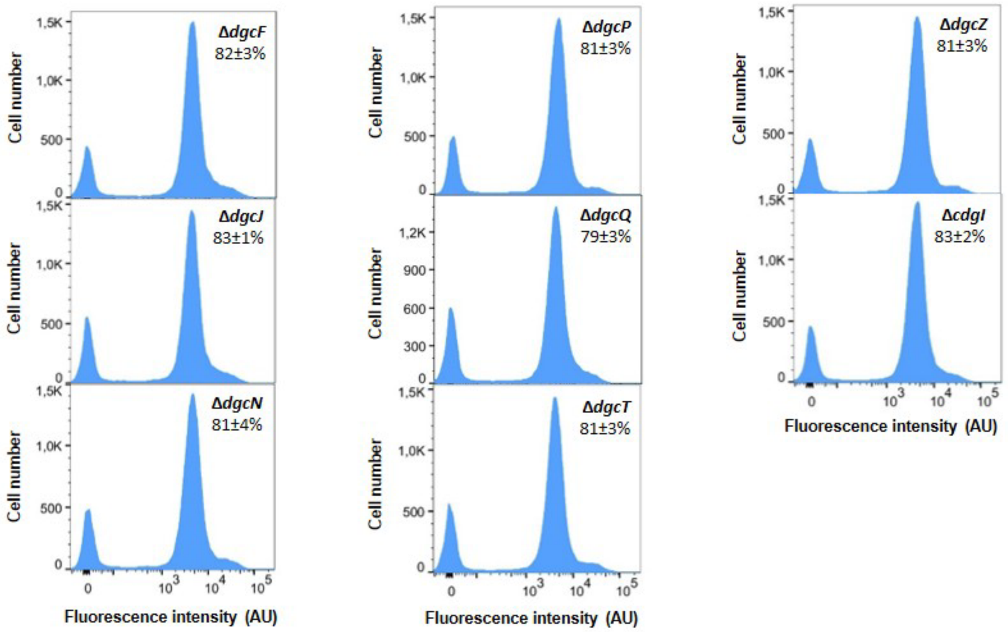
ΔdgcF
82±3%
ΔdgcJ
83±1%
ΔdgcN
81±4%
ΔdgcP
81±3%
ΔdgcQ
79±3%
ΔdgcT
81±3%
ΔdgcZ
81±3%
ΔcdgI
83±2%
Cell number
Fluorescence intensity (AU)

B

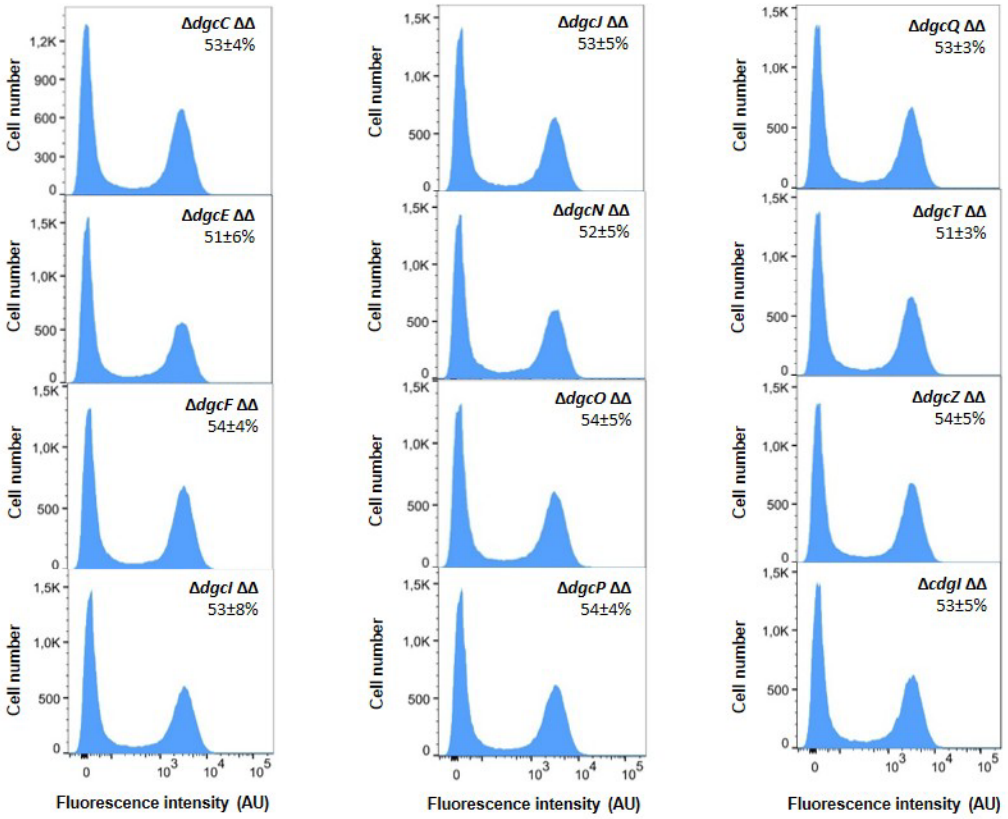
ΔdgcC ΔΔ
53±4%
ΔdgcE ΔΔ
51±6%
ΔdgcF ΔΔ
54±4%
ΔdgcI ΔΔ
53±8%
ΔdgcJ ΔΔ
53±5%
ΔdgcN ΔΔ
52±5%
ΔdgcO ΔΔ
54±5%
ΔdgcP ΔΔ
54±4%
ΔdgcQ ΔΔ
53±3%
ΔdgcT ΔΔ
51±3%
ΔdgcZ ΔΔ
54±5%
ΔcdgI ΔΔ
53±5%
Cell number
Fluorescence intensity (AU)

Figure S11. Deletions of *dgc* genes that do not affect curli gene expression. Gene deletions were introduced either in the WT background **(A)** or in the Δ*dgcM* Δ*pdeR* background (indicated by ΔΔ) **(B)**. Data are for the same *E. coli* cultures as in Figure 4. Fraction of positive cells in the population (mean of at least 3 biological replicates ± SD) is indicated for each strain. Note that the scale of the *y* axes differs between individual strains to improve readability.

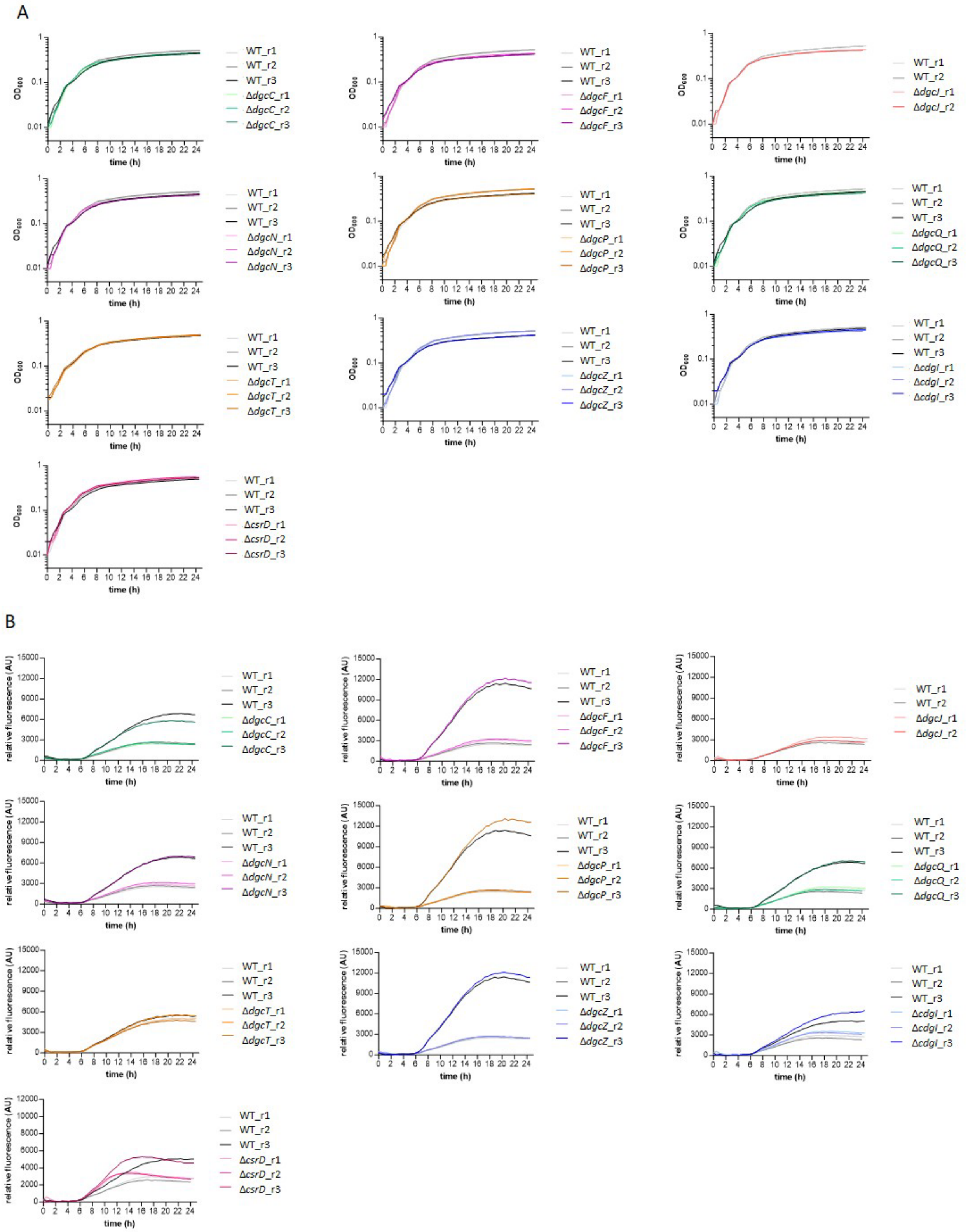
A
OD600
time (h)
WT_r1
WT_r2
WT_r3
ΔdgcC_r1
ΔdgcC_r2
ΔdgcC_r3
ΔdgcF_r1
ΔdgcF_r2
ΔdgcF_r3
ΔdgcJ_r1
ΔdgcJ_r2
ΔdgcN_r1
ΔdgcN_r2
ΔdgcN_r3
ΔdgcP_r1
ΔdgcP_r2
ΔdgcP_r3
ΔdgcQ_r1
ΔdgcQ_r2
ΔdgcQ_r3
ΔdgcT_r1
ΔdgcT_r2
ΔdgcT_r3
ΔdgcZ_r1
ΔdgcZ_r2
ΔdgcZ_r3
ΔcdgI_r1
ΔcdgI_r2
ΔcdgI_r3
ΔcsrD_r1
ΔcsrD_r2
ΔcsrD_r3
B
relative fluorescence (AU)
time (h)

Figure S12. Deletions of *dgc* genes that do not alter curli gene expression throughout growth. *E. coli* cultures were grown in a plate reader as in Figure 2. **(A)** Optical density (OD_{600}) and **(B)** relative fluorescence (absolute fluorescence/OD_{600}) for WT and indicated deletion mutants are shown. r1-r3 refer to 3 independent replicates; in each replicate WT and the indicated deletion mutant were grown in the same experiment.

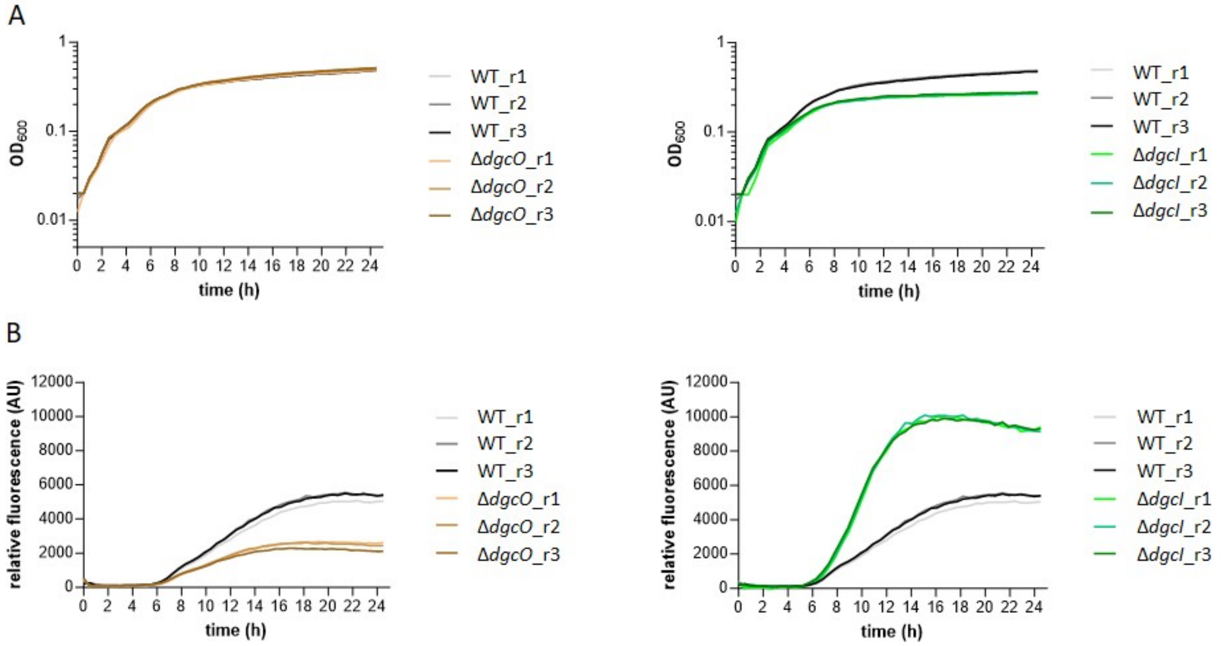

Figure S13. Deletions of *dgc* genes that alter curli gene expression throughout growth. *E. coli* cultures were grown in a plate reader as in Figure 2. **(A)** Optical density (OD_{600}) and **(B)** relative fluorescence (absolute fluorescence/OD_{600}) for WT and indicated gene deletion mutants during growth in a plate reader. r1-r3 refer to 3 independent replicates; in each replicate WT and the indicated deletion mutant were grown in the same experiment.

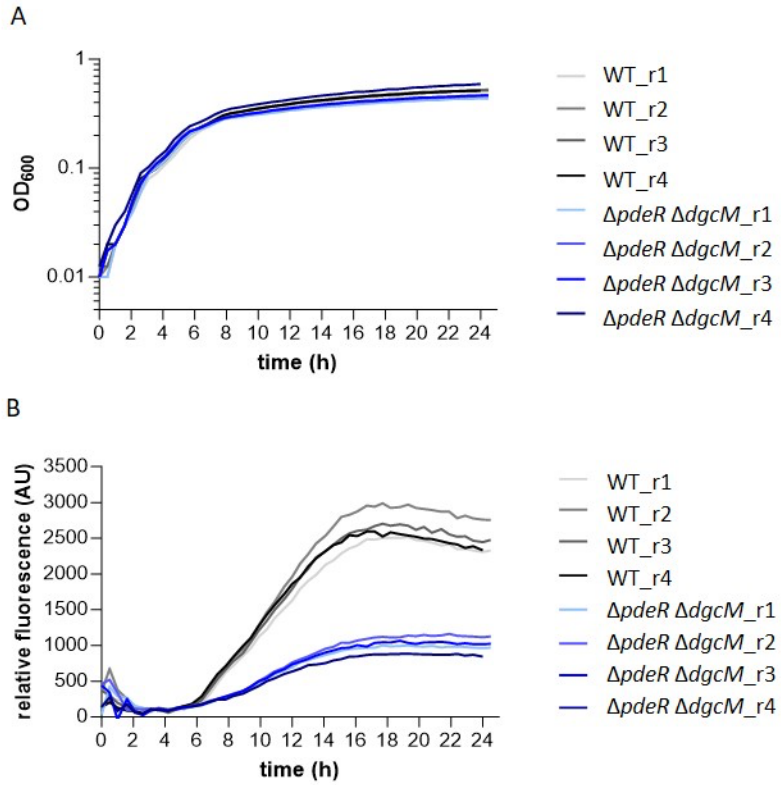

Figure S14. Impact of the *pdeR dgcM* deletion on curli gene expression. *E. coli* cultures were grown in a plate reader as in Figure 2. **(A)** Optical density (OD_{600}) and **(B)** relative fluorescence (absolute fluorescence/OD_{600}) for WT and the Δ*pdeR* Δ*dgcM* strain are shown. r1-r4 refer to 4 independent replicates; in each replicate WT and the Δ*pdeR* Δ*dgcM* strain were grown in the same experiment.

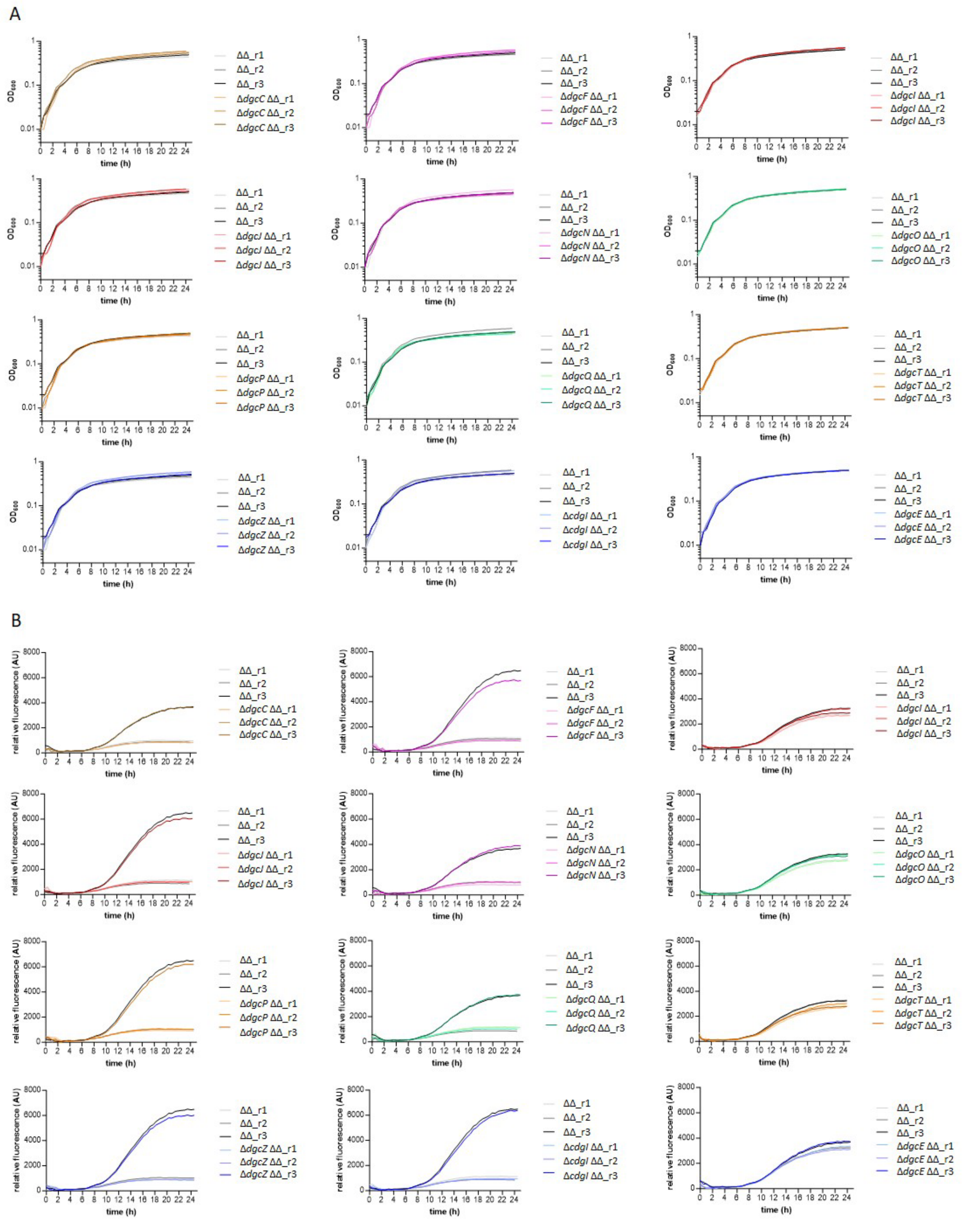

Figure S15. Deletions of *dgc* genes do not alter curli gene expression in the absence of the DgcM/PdeR regulatory module. **(A)** Optical density (OD_{600}) and **(B)** relative fluorescence

(absolute fluorescence/OD_{600}) for the Δ*dgcM* Δ*pdeR* strain (indicated by ΔΔ) and deletions of other *dgc* genes in this background during growth in a plate reader as in Figure 2. r1-r3 refer to 3 independent replicates; in each replicate ΔΔ strain and a corresponding triple gene deletion mutant were grown in the same experiment.

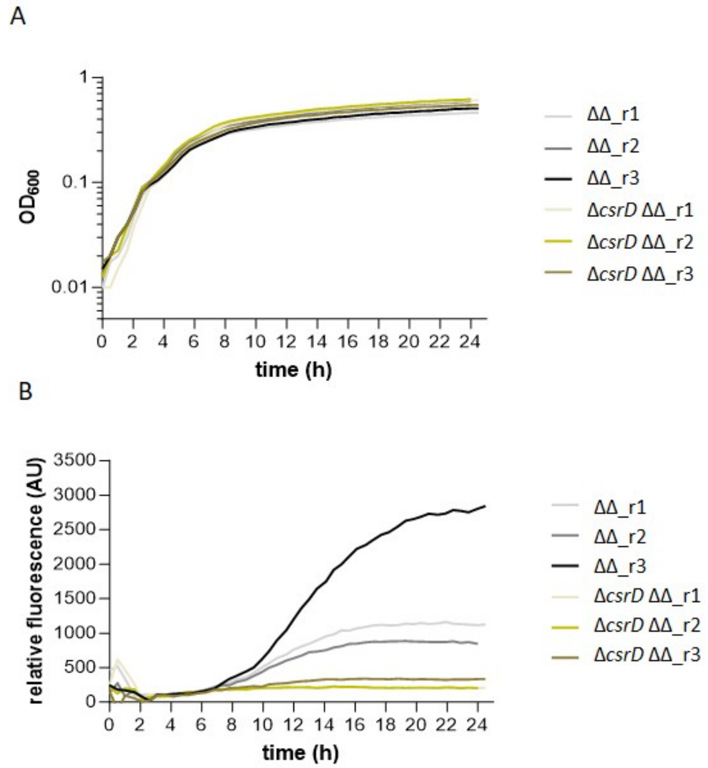

Figure S16. Expression of curli structural genes is strongly reduced upon deletion of *csrD* in the absence of the PdeR/DgcM regulatory module. (A) Optical density (OD_{600}) and **(B)** relative fluorescence (absolute fluorescence/OD_{600}) for the the Δ*dgcM* Δ*pdeR* strain (indicated by ΔΔ) and Δ*csrD* Δ*dgcM* Δ*pdeR* (Δ*csrD* ΔΔ) strain during growth in a plate reader as in Figure 2. r1-r3 refer to 3 independent replicates; in each replicate the ΔΔ strain and a corresponding triple deletion mutant were grown in the same experiment.

Table S1. ***E. coli*** **strains and plasmids used in this study.**

Strains	Relevant genotype	Reference
W3110	W3110 derivative with functional RpoS (Km^S)	[1]
VS1146	W3110 *csgA*::*csgA*-RBS-*sfgfp* (Km^S)	[2]
VS2294	VS1146 Δ*arcA* (Km^S)	This work
VS2295	VS1146 Δ*ariR* (Km^R)	This work
VS2296	VS1146 Δ*basR* (Km^R)	This work
VS2297	VS1146 Δ*bolA* (Km^R)	This work
VS2298	VS1146 Δ*btsR* (Km^R)	This work
VS2299	VS1146 Δ*cdgI* (Km^R)	This work
VS2300	VS1146 Δ*cra* (Km^S)	This work
VS2301	VS1146 Δ*crp* (Km^S)	This work
VS2302	VS1146 Δ*cpxR* (Km^S)	This work
VS2303	VS1146 Δ*csgD* (Km^S)	This work
VS2304	VS1146 Δ*csrD* (Km^S)	This work
VS2305	VS1146 Δ*dgcC* (Km^R)	This work
VS1720	VS1146 Δ*dgcE* (Km^S)	[3]
VS2306	VS1146 Δ*dgcF* (Km^R)	This work
VS2307	VS1146 Δ*dgcI* (Km^S)	This work
VS2308	VS1146 Δ*dgcJ* (Km^R)	This work
VS1257	VS1146 Δ*dgcM* (Km^S)	[3]
VS2309	VS1146 Δ*dgcN* (Km^R)	This work
VS2310	VS1146 Δ*dgcO* (Km^S)	This work
VS2311	VS1146 Δ*dgcP* (Km^R)	This work
VS2312	VS1146 Δ*dgcQ* (Km^R)	This work
VS2313	VS1146 Δ*dgcT* (Km^R)	This work
VS2314	VS1146 Δ*dgcZ* (Km^R)	This work
VS2315	VS1146 Δ*fliA* (Km^S)	This work
VS2316	VS1146 Δ*fliZ* (Km^S)	This work
VS2317	VS1146 Δ*fnr* (Km^R)	This work
VS2318	VS1146 Δ*fur* (Km^S)	This work
VS2319	VS1146 Δ*gadE* (Km^S)	This work
VS2320	VS1146 Δ*hfq* (Km^S)	This work
VS2321	VS1146 Δ*hns* (Km^S)	This work

VS1221	VS1146 Δ*ihfA* (Km^R)	This work
VS2322	VS1146 Δ*iraP* (Km^S)	This work
VS2323	VS1146 Δ*leuO* (Km^R)	This work
VS2324	VS1146 Δ*mqsR* (Km^R)	This work
VS1857	VS1146 Δ*mlrA* (Km^S)	[3]
VS2325	VS1146 Δ*ompR* (Km^S)	This work
VS2326	VS1146 Δ*pdeL* (Km^R)	This work
VS1713	VS1146 Δ*pdeR* Δ*dgcM* (Km^S)	[3]
VS2327	VS1713 Δ*pdeR* Δ*dgcM* Δ*cdgI* (Km^R)	This work
VS2328	VS1713 Δ*pdeR* Δ*dgcM* Δ*csrD* (Km^S)	This work
VS2329	VS1713 Δ*pdeR* Δ*dgcM* Δ*dgcC* (Km^R)	This work
VS1725	VS1713 Δ*pdeR* Δ*dgcM* Δ*dgcE* (Km^R)	This work
VS2330	VS1713 Δ*pdeR* Δ*dgcM* Δ*dgcF* (Km^R)	This work
VS2331	VS1713 Δ*pdeR* Δ*dgcM* Δ*dgcI* (Km^R)	This work
VS2332	VS1713 Δ*pdeR* Δ*dgcM* Δ*dgcJ* (Km^R)	This work
VS2333	VS1713 Δ*pdeR* Δ*dgcM* Δ*dgcN* (Km^R)	This work
VS2334	VS1713 Δ*pdeR* Δ*dgcM* Δ*dgcO* (Km^R)	This work
VS2335	VS1713 Δ*pdeR* Δ*dgcM* Δ*dgcP* (Km^R)	This work
VS2336	VS1713 Δ*pdeR* Δ*dgcM* Δ*dgcQ* (Km^R)	This work
VS2337	VS1713 Δ*pdeR* Δ*dgcM* Δ*dgcT* (Km^R)	This work
VS2338	VS1713 Δ*pdeR* Δ*dgcM* Δ*dgcZ* (Km^R)	This work
VS2339	VS1146 Δ*phoP* (Km^R)	This work
VS2340	VS1146 Δ*qseB* (Km^R)	This work
VS2341	VS1146 Δ*rcdA* (Km^R)	This work
VS2342	VS1146 Δ*relA* (Km^S)	This work
VS2343	VS1146 Δ*rpoS* (Km^S)	This work
VS2344	VS1146 Δ*rscB* (Km^S)	This work
VS2345	VS1146 Δ*rstA* (Km^R)	This work
VS2346	VS1146 Δ*uspE* (Km^S)	This work
VS2347	VS1146 Δ*zraP* (Km^R)	This work
VS2348	VS1146 Δ*hns* Δ*ihfA* (Km^R)	This work
Plasmids		
pTrc99a	Expression vector; *Ptrc* promoter inducible by isopropyl-β-D-thiogalactopyranoside (IPTG); pBR ori ($Ap^{R)}$	[4]

pVS1977	pTrc99a::*csgD* (Ap^R)	This work

Table S2. Primers used in this study to confirm gene deletions.

gene	Forward primer (5'–>3')	Reverse primer (5'–>3')
diguanylate cyclase genes		
cdgI	CGC GAC CAG GAT GAT ATT TAT CC	ACA CTT GCG GCG ATA AAA TGC
csrD	GTC CTG CTG TTC TAA AAG TAA CG	GCT ACG GTG AAA ATT AGT GCA G
dgcC	TGC TAA CGG GTG AGC TAC G	CGT GCT GCA GTG ATC GAA G
dgcF	AAC GAC AAA ATA GCA TTC CCA GG	AGC AAA CTT TAA TAC GCC GAA CC
dgcI	ACT GCG TAA ACA AGG GGT ACG	TGA GTA CGA TTT GTG GGG TAG C
dgcJ	CCT AAA TTT CCC CTC CCC AGC	TAA CCG CTG ATG CGT CAC
dgcN	GCC TGT CAT CAT TCA CAC TCA AC	TGG CAT CTG ATA AGC CCA GC
dgcO	ACC ATA CGA TAT TCG CTG TCA CC	GTA TCC CCA GAG CTT CTC TGC
dgcP	GGT ATC GTT TTG TTT TGC AGC AC	GTA CGA CAA TAA TCG CCA CAC C
dgcQ	CGC GTC TTA TCA TGC CAC C	ATT ACG CGG TGA TTG TGA AAG G
dgcT	TGA TGA AAG GCT GCG CTA CG	GGG AAA TAA TGC GGG CAA CG
dgcZ	GCC TGA ACT ACT GGG CCA TTA GG	GGG CCT GAT TTT TAT GAC GGC GA
other regulator genes		
arcA	GGA AAG TGC ATC AAG AAC GCA A	CAC TGC CGA AAA TGA AAG CCA G
ariR	GCA ATA GTG ATT CAG GAG GGC CA	CGT CAT GAA CAC CTC CCT CTC GA
basR	CCA GAA CTG CCT GCA AAA ACA G	GCT GAT CAG CTC AAA CAC CAA C
bolA	CGC TTG ACG GAA AAA CCA GG	ATT TTC GTC TGA ATG CTG GGC A
btsR	TGG GGA TGA ATC TGG TGG ATA AGC	GCG GTG GCT TCG ACA TTA CC
cpxR	GAG AGT TTA CGA TTC AGG CTG CA	ACG CTG TTC GCT ATC CAG AAG
cra	AGT GGG AAC CTG GAA TAA AAG CAG	TCA TCG CCG CAT GTA ACA AAA
crp	TTT GCT ACT CCA CTG CGT CAA TTT T	CTT TTG CCA GTA GTA GCT GTG TCA G
csgD	TTT TAC ATG ACG AAA GGA CTA CAC C	GAT TGA CCG TTA TAG TGA TCC AGC
fliA	AGC GCA ATT TGT CAG CAA CG	GCG ATC GAG TAA TTT ACG GCA A
fliZ	AGC GCG AAA AAC TGG TAT TAA CC	ATC ACA CCC ATC AAT GCC TGA C
fnr	GGT TTG CTT AGA CTT ACT TGC TCC C	TCC TTA CAA CAA CTG TCA ACG CAG
fur	GCC TTG CCG TTG TAA ATG TAA GC	GGA AAT GAT CAG GCG GTG AAA GC
gadE	GAG TGC GTG ATG GAT AAA TCT GAA G	GGT GGA TAC ACA TAC CAG GGG AA
hfq	TTC GTT GCG TGG GTT ATC GCC AGA TG	AGC ACC CGT CGC TTT GAC AGC TTC
hns	TGG CGG CAC AAA ATA AAG AAC AA	CGT ATC GGT GTT ATC CAC GAA AC

ihfA	TTG CGG AGG GGT ATA AGA GC	CAG CAG CAT CAA AAC TGA CAG G
iraP	CCT AAA TGG CCA TGC GGT GAG	GTG TCC GGG CTT TTG TCA CAG
leuO	GAC TCA TTC CAC GGC AAT GGA	TCG GCT GAA TCC CAC AAC TTA CA
mlrA	TTA AAA CGC GTA ACA TAC AT	CAG ACC GCC TGT TTG AAA GG
mqsR	CGT GAT GCC TGA CTC CAG C	TGC TCT CTT CGC AAT GGA CAC
ompR	GCG CAC ATT GGG TAT AAC GTG	TGC AGT TTG TCG GTC ATC AAC A
pdeL	TGA GCG AGC ATC AGA GGT AAA G	CGG GAG AAT TAA TCG CTG CCA
phoP	AAC CTC GTA TCA GTG CCG GA	GAT TGC TCT CGC CAC GTA ACA
qseB	CAT TAC TGC GAT TAC TGC TGC GA	AGC ATC AGT TGG GTG TCG AAC
rcdA	GAA GAA CAT AAA CAG CGC CCA G	CAG AAC TTT TCC GCA GGG CA
rcsB	CGC TGG AAC ATC TGA TTC GTG AG	AGT GCA AAT GCC AGA TGC GA
relA	TGG CTC GGG ATA GCG AAG C	CAA CGC TTT ACG CTA CTG TGG A
rpoS	ACC GGA ACC AGT TCA ACA CG	TTC TTA ATT ACC TGG TGC GTA TGG G
rstA	ACA CCA ATC AAC AGC ACT ACC A	TGC GCA TCA GAT ACA GCG AAC
uspE	ACG TTG CCT GAT TTT TCC GCA	ATT CCG CTG TCT GAG TGG AC
zraP	GCC ATC TTT TAT CAG CGC TTA CC	GCG AAG GGC AAA CCA TTA AAC C

Supplemental References

1. Serra DO, Richter AM, Klauck G, Mika F, Hengge R. Microanatomy at cellular resolution and spatial order of physiological differentiation in a bacterial biofilm. mBio. 2013;4(2):e00103-13. Epub 2013/03/21. doi: 10.1128/mBio.00103-13.
2. Besharova O, Suchanek VM, Hartmann R, Drescher K, Sourjik V. Diversification of gene expression during formation of static submerged biofilms by *Escherichia coli*. Frontiers in Microbiology. 2016. doi: 10.3389/fmicb.2016.01568.
3. Lamprecht O, Ratnikava M, Jacek P, Kaganovitch E, Buettner N, Fritz K, et al. Regulation by cyclic di-GMP attenuates dynamics and enhances robustness of bimodal curli gene activation in *Escherichia coli*. PLOS Genetics. 2023;19(5):e1010750. doi: 10.1371/journal.pgen.1010750.
4. Amann E, Ochs B, Abel K-J. Tightly regulated tac promoter vectors useful for the expression of unfused and fused proteins in *Escherichia coli*. Gene. 1988;69(2):301-15. doi: https://doi.org/10.1016/0378-1119(88)90440-4.
